# Cooperation patterns of members in networks during co-creation

**DOI:** 10.1038/s41598-021-90974-1

**Published:** 2021-06-08

**Authors:** Kunhao Yang, Itsuki Fujisaki, Kazuhiro Ueda

**Affiliations:** 1 Graduate School of Arts and Sciences, The University of Tokyo, Tokyo, 153-8902 Japan; 2 Research Fellowship for Young Scientists (DC2), Japan Society for the Promotion of Science (JSPS), Tokyo, 102-0083 Japan

**Keywords:** Social behaviour, Complex networks

## Abstract

Cooperation (i.e., co-creation) has become the principal way of carrying out creative activities in modern society. In co-creation, different participants can play two completely different roles based on two different behaviours: some participants are the *originator*s who generate initial contents, while others are the *revisor*s who provide revisions or coordination. In this study, we investigated different participants’ roles (i.e., the originator *vs.* the revisor) in co-creation and how these roles affected the final cooperation-group outcome. By using cooperation networks to represent cooperative relationships among participants, we found that peripheral members (i.e., those in the periphery of the cooperation networks) and core members (i.e., those in the centre of the cooperation networks) played the roles of originators and revisors, respectively, mainly affecting the quantity versus the quality of their creative outcomes. These results were robust across the three different datasets and the three different indicators defining core and peripheral members. Previous studies have considered cooperation behaviours to be homogeneous, ignoring that different participants may play different roles in co-creation. This study discusses patterns of cooperation among participants based on a model in which different roles in co-creation are considered. Thus, this research advances the understanding of how co-creation occurs in networks.

## Introduction

Creative activity is regarded as a key factor in the development of modern society^1^. Many previous studies^2–7^ have found that as society becomes increasingly complex, cooperation rather than individual effort has become the main way of carrying out creative activities. In this regard, it is important to understand how cooperation (i.e., co-creation) occurs among participants during creative activities. Many previous studies^8–18^ have discussed this issue by focusing on the network among the participants in cooperation (co-creation). Specifically, previous studies^8–12^ in network science have discussed how the network structure leads to cooperation in a *prisoner’*s *dilemma* game (PD). In these studies, all participants in the PD constituted a network^19^. In each round of the PD, a focal participant and one of the network neighbours (i.e., another participant connected to the focal participant in the network) were randomly selected for interaction. In the interaction, the two participants could choose one of two strategies: to cooperate or defect. The two participants’ payoffs were then determined differently according to their chosen strategies. Since in the PD, regardless of one’s neighbour’s choice, defection can always provide the focal participant with a higher payoff, it is natural to assume that defection will become the dominant strategy after a sufficiently large number of interactions^19^. However, previous studies^8–12^ found that if there was a *core-periphery structure* in the network, the cooperation strategy could survive in the PD. In a network with a core-periphery structure, there are two types of participants: *core* members and *peripheral* members^20^. The core members are defined as well-connected, and they also make up the information hub of the network^13–18,20,21^. In contrast, peripheral members are defined as those with few connections who are far from the information hub^13–18,20,21^. Based on these definitions, previous studies^8–12^ have found that the existence of core members in a network may lead to the survival of the cooperation strategy in PD. To be more specific, Santos and Pacheco^8^ found that within a scale-free network in which a small group of core members is connected to an extremely large number of peripheral members, the cooperation strategy could become dominant in the PD after a large number of interactions. Subsequent studies^9–11^ then pointed out that the greater influence core members have on the whole network and﻿ the shortcuts connected to the core members were keys to the survival of cooperation strategy in the PD. Finally, a recent study^12^ also pointed out that the above results were robust even considering a PD with high-order interactions (i.e., in each round, the interaction occurred among more than two participants).

Previous studies^13–18^ examining creative activities have also focused on the core-periphery structure in the network. They compared the creative outcomes generated by core and peripheral members in co-creation. In these studies, co-creation was considered to comprise co-submitting behaviours of original contents (co-submissions of new ideas, software code, scientific papers, etc.); they then constructed networks based on the co-submission relationships (e.g., the co-author relationship in research-paper-writing activities) among participants. Based on these cooperation networks, the participants were divided into groups of core and peripheral members. These previous studies found that, contrary to what might be intuitive, peripheral members made larger contributions to creative outcomes than core members in co-creation activities. In other words, most of the original content was submitted by peripheral rather than core members in co-creations.

**Figure 1:**
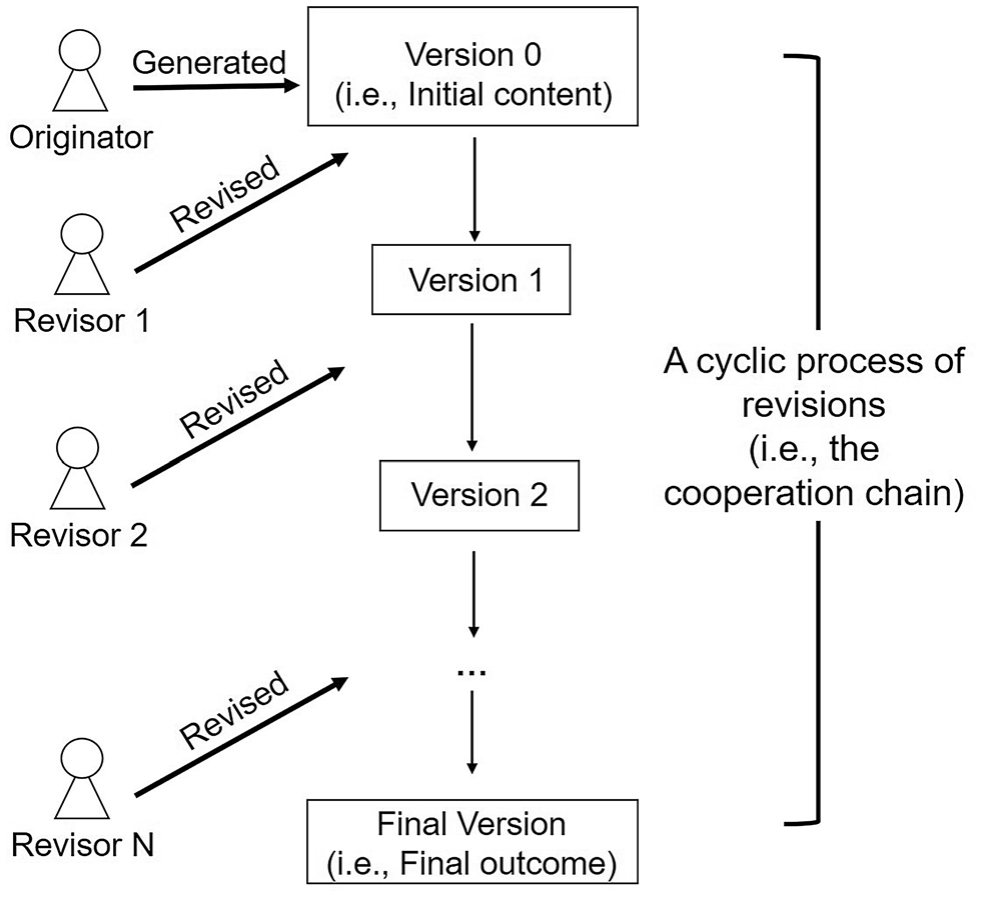
The different roles in co-creation. Cooperation is a cyclic process of revisions: an originator submits the initial content while other participants (i.e., the revisors) provide revisions and generate new versions of the content in turn. The final output of the cooperation chain is the final version of the content.

In summary, the above two types of previous studies^8–18^ emphasised the importance of the core-periphery structure of networks for co-creation (cooperation). However, in these studies, different participants’ cooperation behaviours (strategies) were considered to be *homogeneous*. Specifically, in PD, there was only one type of cooperation strategy. Therefore, if participants A and B chose to pursue the cooperation strategy in the PD, their cooperation behaviours were assumed to be the same. Similarly, in the case of co-submissions, all participants in the cooperation were assumed to carry out the same behaviour: original content submission. However, in the real world, different participants often engage in *heterogeneous* (i.e., different) behaviours in cooperation. In this regard, previous studies^22,23^ have pointed out that co-creation is a *cyclic* (i.e., repeating) *process of revisions*; after the initial contents are submitted by some participants, these contents always needed several revisions by other participants to produce the final outcome (see the illustration in Fig. 1). As a result, different participants may engage in two completely different behaviours through their different roles in co-creations^24–28^. Some participants were the *originator*s who generated initial content, while others were the *revisor*s who provided revisions or coordination. Therefore, in order to understand co-creation among different participants, it is not sufficient to focus on those who cooperated and those who did not (i.e., defected in the PD). The more important issues are what roles (i.e., the originator *vs.* the revisor) the different participants play in co-creation and how these roles affect the final outcome of the cooperation group or community.

Based on the above discussions, this study revisits the issue of co-creation and focuses on the different roles of participants in these activities. Specifically, based on previous studies^8–18^, we also focus on the core and peripheral members in networks and compare their different roles. In the following sections, we empirically analyse three different co-creation activities represented by three different datasets. In particular, we seek to answer two important questions: 1) what different roles (i.e., originator and revisor) do the different participants (i.e., the core members and peripheral members) play, and 2) how do these roles affect the final outcome of the co-creation? By analysing different participants’ roles in co-creation, this study provides a deeper understanding of the cooperation pattern among participants in networks.

## Results

### Overview of the data and measurement of the core-peripheral position

To investigate the participants’ different roles in co-creations, we used three datasets collected from websites: 1) SCP-Wiki, representing story-writing activities^29^, 2) GitHub, representing software development activities^30^, and 3) Idea Storm, representing idea generation activities^31^ (see details in the “Materials and methods” section). In all the datasets, the final outcomes (i.e., SCP-stories in SCP-Wiki, source codes in GitHub, and ideas in Idea Storm) were generated through a cooperation chain of participants: an originator submitted the initial content and the revisors provided revisions and generated the next versions of the content; the final outcome was the final version of the content (see Fig. 1).

Cooperation networks must first be constructed in order to define the core-periphery positions. Based on the results of previous studies^13–18,32–40^, we constructed cooperation networks through cooperation relationships in our three datasets. The participants who worked on the same content (i.e., the same SCP-stories, source codes, or ideas) were assumed to be connected in their respective networks (i.e., undirected weighted networks; the weight of the connections indicates the times of cooperation between the two members). Based on previous research^13–18,32–40^, we used three metrics (i.e., core-periphery metric hereafter) to measure the core-periphery positions of participants in the manuscript: *degree*, *k-core*, and *eigenvector centrality* (see the details of computations in the “Materials and methods” section). Degree^13–18^ indicates whether the focused node (i.e., participants) is well-connected. A larger degree value indicates that the focused node has more connections with the other nodes in the network. K-core^35–38^ indicates whether the node is in a cohesive subgroup (i.e., a subgroup with many connections among its members). A large k-core value reflects a large number of connections between the focused node and its neighbours (i.e., nodes with connections with the focused node). Eigenvector centrality^32–34,38–40^ indicates whether the focused node is the information hub of the network. A large value of eigenvector centrality indicates that the focused node is at the centre of the information flow of the network. In this study, nodes with larger values of degree, k-core, or eigenvector centrality were regarded as closer to the core of the network. In the following analysis, we show that our results are robust across these three metrics. Additionally, in the following analyses, we compare the values of our three core-periphery metrics at different time points to identify the core (peripheral) members at different time points. However, the features (e.g., density) of the networks at different time points will also affect the values of the core-periphery metrics and may make them incomparable. Therefore, we normalised our three metrics over time to ensure that their values can be compared between different time points (see details in the “Materials and methods” section).

In addition to the above three core-periphery metrics in the manuscript, in the ‘S4’ section in ‘Supplementary Information’, we also used another core-periphery metric: betweenness centrality^41,42^. This indicator measures how many shortcuts (i.e., the shortest path between a pair of nodes; see details in the ‘S4’ section in ‘Supplementary Information’) in the network passed the focal node^41,42^. Simply speaking, a node with a large betweenness centrality can be considered as an information hub^41^, namely, a core member. Note that, the details of results based on betweenness centrality were only shown in ‘Supplementary Information’ because many previous studies have pointed out that although the betweenness centrality is a very important metric for detecting subgroups in networks (i.e., for community detections)^42^, it is not as effective as the eigenvector centrality^38–40^ and k-core^38^ for identifying the specific core members (i.e., nodes). In the remaining parts of the manuscript, therefore, the results based on betweenness centrality are reported briefly.

### Role division of core and peripheral members

**Figure 2:**
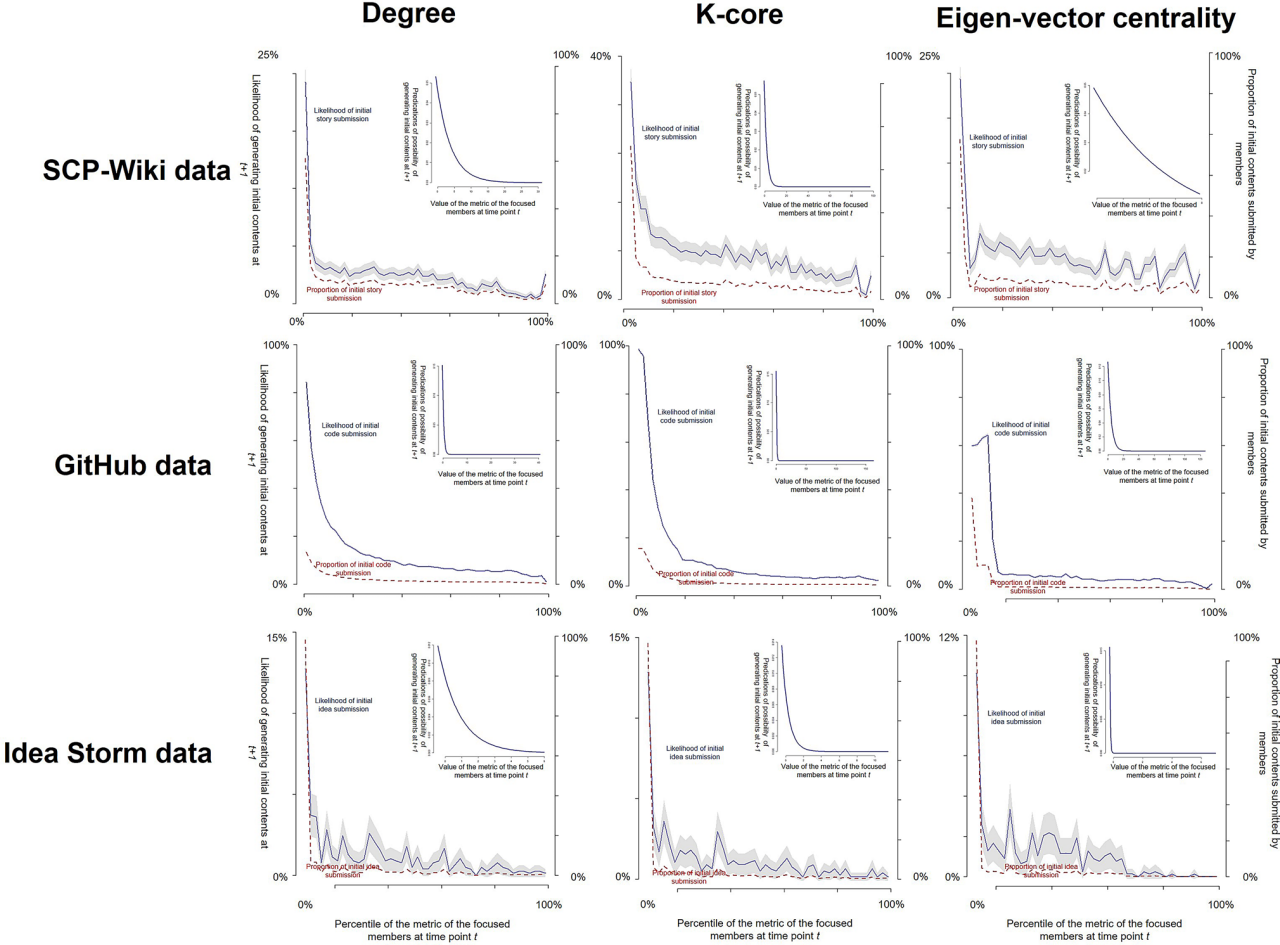
The relationship between the values of the core-periphery metrics and the initial content submissions. In this figure, the relationships between the different values of the core-periphery metrics and the initial content submission are shown for SCP-Wiki, GitHub, and Idea Storm. The panels in the same row show the results for the same dataset using different metrics (i.e., degree, k-core, eigen-vector centrality). The panels in the same column show the results using the same metric but for different datasets. For each panel, the *x*-axis indicates the percentile of values of the core-periphery metrics at time point *t*. The left *y*-axis indicates participants’ likelihood of submitting initial content at time point *t* + 1. The right *y*-axis indicates the proportion of initial contents submitted by the participants to all the initial contents in the communities. The red lines corresponding to the right *y*-axis show the percentages of initial contents submitted by participants with different values of the core-periphery metrics. The blue lines corresponding to the left *y*-axis show the percentages of participants with different values of core-periphery metrics that would submit original content at the next time point. The grey area shows the 95% confidence intervals of the blue lines, generated by two-tailed *t*-tests (note that the confidence intervals for the GitHub data are too narrow to be seen). The insets show the predicted possibilities of initial content submissions by the participants who had different values of core-periphery metrics but shared the same number of days in the communities (equalling the average number days that all participants spent in the communities). The predicted possibilities of initial content submissions were generated by a logistic model with the likelihood of initial content submissions as the dependent variable, the values of the core-periphery metrics as the independent variable, and the number of days that a participant spent in the communities as the control variable.

First, we investigated who played the role of originator (revisor) in co-creation. The red lines in Fig. 2 show the percentages of the total initial content submitted by participants with different values of core-periphery metrics. The results were consistent across all three datasets regardless of which of the three metrics we used: participants with smaller values of core-periphery metrics (i.e., peripheral members) submitted a larger proportion of initial content, while participants with larger values of core-periphery metrics (i.e., core members) contributed less initial content. Therefore, compared to the core members, the peripheral members seemingly played a more important role in the initial content submissions. However, these results can be easily explained by the fact that there are far more peripheral members than core members in the networks. Therefore, we then examined the relationship between the percentiles of the core-periphery metrics at time point *t* and the likelihood of submitting the initial content at time point *t* + 1. This relationship reflects the *possibility* of one core (peripheral) member submitting initial contents at the next time point. The blue lines in Fig. 2 show that in all three datasets, the participants with a smaller value of core-periphery metrics at time point *t* had a significantly larger likelihood of submitting initial contents at time point *t* + 1. In other words, when a participant was a peripheral member, they were more likely to submit initial contents, while when they became a core member, they were less likely to submit initial contents. Nevertheless, there is still an alternative explanation for the above results: the peripheral members were more likely to submit initial contents because they were newcomers. In other words, most of the peripheral members are newcomers, and if a newcomer does not get a chance to create initial content, they may simply choose to leave the community; therefore, the relationship between the core-periphery positions and the possibility of initial content submission can only be explained by the time factor. To exclude this alternative explanation, we used logistic models to control for the number of days that a participant had spent in the community. In other words, the logistic models employed the likelihood of initial content submissions as the dependent variable, the values of the core-periphery metrics as the independent variable, and the number of days that a participant had spent in the community as the control variable. The insets in Fig. 2 show the predicted possibilities of initial content submissions for the participants who had different values of core-periphery metrics but shared the same number of days in the communities (which equals the average number of days for which all participants spent in the communities). The results indicate that even after controlling for the time factor, the peripheral members still have a significantly larger possibility of submitting initial contents at the next time point than core members. These results were consistent across all datasets using all the different metrics. In the ‘S4’ section and Fig. S6 in ‘Supplementary Information’, we showed that the results across all datasets using the betweenness centrality were also consistent with the results in Fig. 2.

In summary, the above results showed that in co-creations, the peripheral members mainly played the role of originator and the core members mainly played the role of revisor. However, these results were only based on the quantity (i.e., possibility) of the initial content submissions. Another question then arises regarding content quality: how did one’s position as a core (or peripheral) member affect the content quality by role? In particular, previous research^26–28^ found that core members often have more professional knowledge of creative activities than peripheral members. Therefore, if core members play the role of originator (or revisor) in co-creation activities, can they significantly benefit the quality of the final outcome? To address this issue, we analyse the quality of the final outcomes for the three datasets in the next section.

### Quality of original content submitted by peripheral and core members

We employed participant-generated quality metrics, *rate* and *star*, to measure the quality of the final outcomes (i.e., SCP-stories and codes) in the SCP-Wiki and GitHub data, respectively. Both indicate how many other participants in the communities thought that the SCP-stories or codes were good (see details in the “Materials and methods” section). In Idea Storm, we used a specialist-generated metric to measure the quality of ideas, namely, whether or not the team of specialists at Dell (the owner of the Idea Storm website) thought that the submitted ideas were valuable (see details in the “Materials and methods” section). We then examined the relationships between the percentiles of the core-periphery metrics of originators at *t* and the quality of the final outcomes that they submitted at *t* + 1. The quality of final outcomes was measured by (a) the average rate of the stories in SCP-Wiki*,* (b) the average number of stars of the code repertories in GitHub, and (c) the proportion of valuable ideas in Idea Storm.

**Figure 3:**
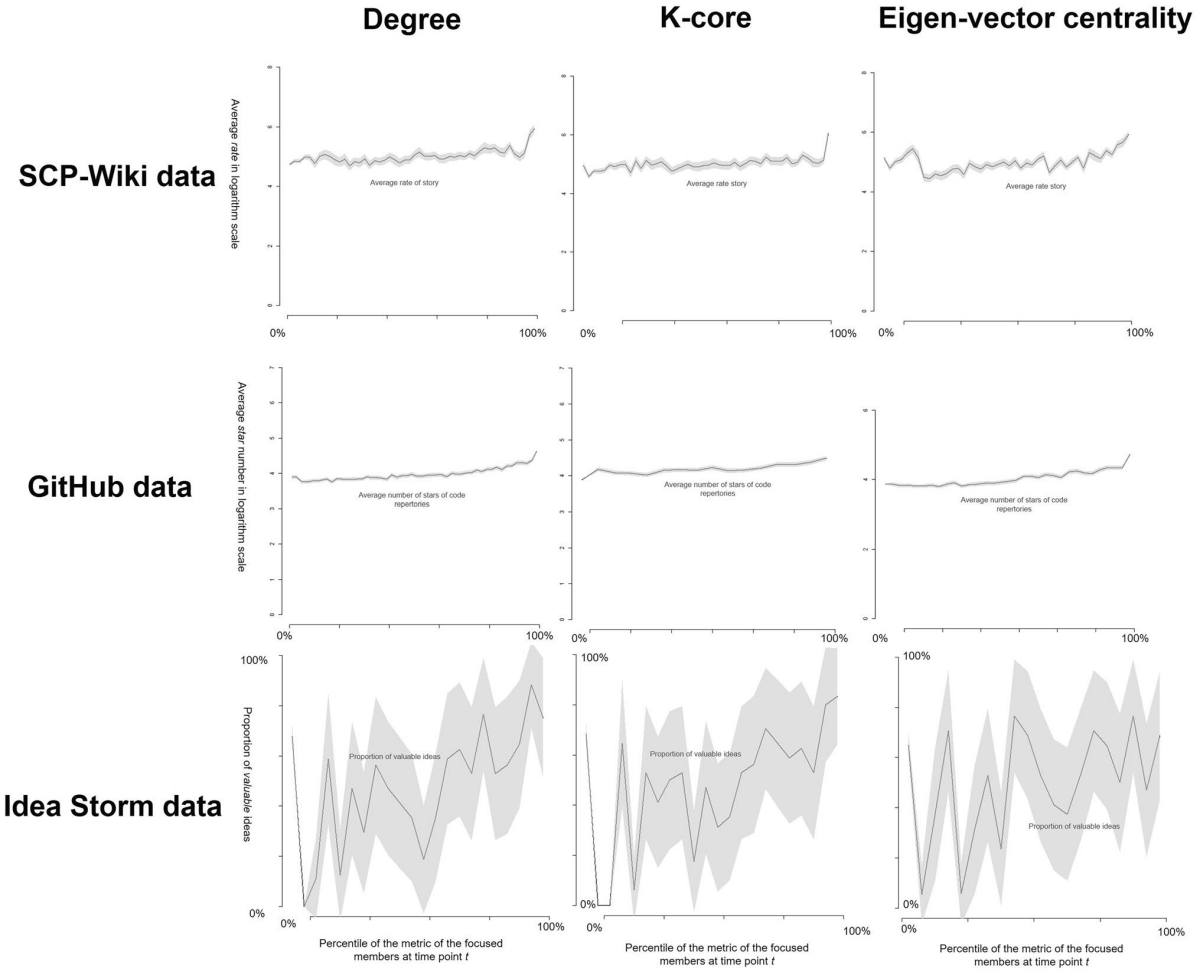
The relationship between the values of the core-periphery metrics and the quality of final outcomes. In this figure, the relationships between the different values of the core-periphery metrics and the values of the content-quality metrics are shown for SCP-Wiki, GitHub, and Idea Storm. The panels in the same row show the results for the same dataset using different metrics (i.e., degree, k-core, eigenvector centrality). The panels in the same column show the results using the same metric for different datasets. For each panel, the *x*-axis indicates the percentile of values of the core-periphery metrics at time point *t*. The *y*-axis indicates the value of each quality metric of the content; in SCP-Wiki data, it represents the average rate on a logarithmic scale; in GitHub data, it represents the average star number on a logarithmic scale; in Idea Storm data, it represents the average proportion of valuable ideas. The lines show the relationship between originators’ values of the core-periphery metrics and the average quality of the final outcomes. The grey area shows the confidence intervals of the average value generated by the two-tailed *t*-test (note that the confidential intervals in GitHub data were too narrow to be seen). In the Idea Storm data, the proportion of valuable ideas fluctuated, probably because most participants only submitted a small number of ideas (e.g., one or two ideas), in which case even only one idea was evaluated as valuable. This inevitably generated a large fluctuation (e.g., from 0 to 50%) in the proportion of valuable ideas.

The lines in Fig. 3 show that the value of the quality metrics slightly increased when the values of the core-periphery metrics increased. Fig. S7 in ‘Supplementary Information’ shows that the same results were observed across all datasets using betweenness centrality. These results seemingly indicate that the final outcomes originating from the core members were of higher quality. However, we considered that the higher quality could also be caused by subsequent revisions because previous research^26–28^ indicates that the contents that had originated from the core members easily received more revisions by other core members.

To control for the influence of revisors and other related factors, we used regression models considering five important control variables^26–28^. We controlled 1) the average value of the core-periphery metrics of the revisions; 2) the number of content revisions; 3) the time point of the initial content publication; 4) the number of days from the originator’s first participation (i.e., submission or revision) until now (which indicated the days the originator was observed), and 5) the originator’s number of previous participations. Control variables 4) and 5), which indicated that previous experience of the originators on the websites, were added because previous research^26–28^ found that the quality of outcomes from very experienced participants (i.e., originators) in the communities (i.e., the websites) tended to be overestimated. In contrast, the quality of outcomes for inexperienced participants in the communities tended to be underestimated. By controlling all the related factors, the results of the regression models (in Tables 1, 2, 3) showed that the values of the core-periphery metrics of the originators did not have a significant positive effect on content quality (i.e., the three core-periphery metrics did not have significant positive coefficients in any of the regression models; see the specific coefficients in the fourth row of Tables 1, 2, 3). In Tables S3–S5 in ‘Supplementary Information’, we show that the regression results based on the betweenness centrality are also consistent with the results in Tables 1, 2, 3. These regression results indicate that regardless of their greater corresponding professional knowledge, core members with higher values of core-periphery metrics did not benefit the quality of the final outcome through their role as **originators** in co-creation.

**Table 1:** Results of the regression of content quality for SCP-Wiki data; coefficients of the core-periphery metrics (i.e., the independent variable) are shown in the fourth row.

Dependent variable: rate in logarithm scale in SCP-Wiki					
Core-periphery metric: degree		Core-periphery metric: k-core		Core-periphery metric: eigen-vector centrality	
Variable	Coefficient	Variable	Coefficient	Variable	Coefficient
Degree of originator in logarithm scale	0.027 (0.021)	K-core of originator in logarithm scale	0.008 (0.019)	Eigen-vector centrality of originator in logarithm scale	0.002 (0.018)
Average degree of revisors in logarithm scale	0.275*** (0.030)	Average k-core of revisors in logarithm scale	0.183*** (0.028)	Average eigen-vector centrality of revisors in logarithm scale	0.097*** (0.020)
Number of content revisions	0.293*** (0.014)	Number of content revisions	0.285*** (0.014)	Number of content revisions	0.266***(0.014)
Time point of publication	0.027 (0.017)	Time point of publication	0.040** (0.017)	Time point of publication	0.057*** (0.016)
Days the originator was observed	− 0.062*** (0.016)	Days the originator was observed	− 0.035** (0.016)	Days the originator was observed	− 0.028* (0.015)
The originator’s number of previous participations	− 0.064** (0.029)	The originator’s number of previous participations	− 0.164*** (0.027)	The originator’s number of previous participations	− 0.286*** (0.016)
Constant	− 0.000 (0.013)	Constant	− 0.000 (0.013)	Constant	− 0.000 (0.013)
*R*^2^	0.247	*R*^2^	0.239	*R*^2^	0.237
Observations: 4653					

*p < 0.1; **p < 0.05; ***p < 0.01; Standard Error is shown in ( ); since all coefficients in the table were estimated based on the standardized variables, the sizes of the coefficients are comparable.

**Table 2:** Results of the regression of content quality for GitHub data; coefficients of the core-periphery metrics (i.e., the independent variable) are shown in the fourth rows.

Dependent variable: number of stars in logarithm scale in GitHub					
Core-periphery metric: degree		Core-periphery metric: k-core		Core-periphery metric: eigen-vector centrality	
Variable	Coefficient	Variable	Coefficient	Variable	Coefficient
Degree of originator in logarithm scale	− 0.008 (0.005)	K-core of originator in logarithm scale	− 0.025*** (0.005)	Eigenvector centrality of originator in logarithm scale	− 0.008** (0.004)
Average degree of revisors in logarithm scale	0.100*** (0.004)	Average k-core of revisors in logarithm scale	0.108*** (0.005)	Average eigen-vector centrality of revisors in logarithm scale	0.017*** (0.004)
Number of content revisions	0.474*** (0.003)	Number of content revisions	0.472*** (0.003)	Number of content revisions	0.486*** (0.003)
Time point of publication	− 0.013*** (0.004)	Time point of publication	− 0.008** (0.004)	Time point of publication	0.011*** (0.003)
Days the originator was observed	− 0.002 (0.003)	Days the originator was observed	0.004 (0.003)	Days the originator was observed	0.001 (0.003)
The originator’s number of previous participations	0.059*** (0.004)	The originator’s number of previous participations	0.068*** (0.005)	The originator’s number of previous participations	0.011*** (0.003)
Constant	0.00000 (0.003)	Constant	0.00000 (0.003)	Constant	0.00000 (0.003)
*R*^2^	0.242	*R*^2^	0.240	*R*^2^	0.236
Observations: 99,232					

*p < 0.1; **p < 0.05; ***p < 0.01; Standard Error is shown in ( ); since all coefficients in the table were estimated based on the standardized variables, the sizes of the coefficients are comparable.

**Table 3:** Results of the regression of content quality for Idea Storm data; coefficients of the core-periphery metrics (i.e., the independent variable) are shown in the fourth rows.

Dependent variable: idea is valuable or not in in idea storm					
Core-periphery metric: degree		Core-periphery metric: k-core		Core-periphery metric: eigen-vector centrality	
Variable	Coefficient	Variable	Coefficient	Variable	Coefficient
Degree of originator in logarithm scale	− 0.549*** (0.154)	K-core of originator in logarithm scale	− 0.845*** (0.156)	Eigenvector centrality of originator in logarithm scale	− 0.029 (0.072)
Average degree of revisors in logarithm scale	5.631*** (0.425)	Average k-core of revisors in logarithm scale	5.436*** (0.397)	Average eigen-vector centrality of revisors in logarithm scale	1.569*** (0.157)
Number of content revisions	0.350* (0.186)	Number of content revisions	0.348** (0.171)	Number of content revisions	0.210** (0.102)
Time point of publication	− 0.334* (0.184)	Time point of publication	− 0.352* (0.198)	Time point of publication	− 0.782*** (0.256)
Days the originator was observed	− 0.029 (0.120)	Days the originator was observed	− 0.024 (0.120)	Days the originator was observed	− 0.037 (0.091)
The originator’s number of previous participations	0.198 (0.192)	The originator’s number of previous participations	0.195 (0.186)	The originator’s number of previous participations	− 1.352*** (0.209)
Constant	0.405*** (0.128)	Constant	0.590*** (0.128)	Constant	0.209* (0.118)
AIC	462.734	AIC	473.470	AIC	783.515
Observations: 837					

*p < 0.1; **p < 0.05; ***p < 0.01; Standard Error is shown in ( ); since all coefficients in the table were estimated based on the standardized variables, the sizes of the coefficients are comparable.

Additionally, a more important point in the regression results is that the average value of the core-periphery metrics of the revisions had a significant positive effect on the quality of the final outcome (see the specific coefficients in the fifth row of Tables 1, 2, 3). Moreover, in Tables S3–S5 in ‘Supplementary Information’, the same results were observed across all datasets using betweenness centrality. These results indicate that, compared to those with small values of core-periphery metrics (i.e., revisors who were peripheral members), the revisions with large values of core-periphery metrics (i.e., revisors who were core members) were those who mainly improved the quality of the final outcome. In other words, this result indicated that the core members benefited from the quality of the final outcome through taking the role of **revisors** in co-creation. Based on these results, an important question regarding what the revisions from the core members changed was resolved. We investigate this question in the next section.

### Core members’ revisions contribute to the integration of the writing style of different stories

To examine the effect of revisions on content changes, we focused only on the SCP-Wiki data. Because SCP-Wiki provides a unique *revising history* function, we can compare the revised parts of different versions of SCP-stories. Using this function, we gathered the parts of the SCP-stories that changed during each revision. To measure the differences between two versions of SCP-stories, we used a document-embedding model^43^ based on a powerful representation model for language, BERT^44^, to project each version of the SCP-stories onto a 768-length vector. The relationships among these vectors can reflect the relationships among the corresponding versions of SCP-stories^43–45^: a larger cosine distance of the vectors represents a smaller similarity between the versions (see details in the “Materials and methods” section). We then computed the average cosine distances between the focal version and all other versions of all SCP-stories in SCP-Wiki. This average cosine distance indicates the focal version’s distance to the centre of all stories in the SCP-Wiki community. We then traced the change in the cosine distance caused by the revisions. We computed the change in the cosine distance between *k* times and *k* − 1 times versions of the same story:

$$\Delta Cosine Distance_{t_k}^{\theta } =Cosine Distance_{t_k}^{\theta }-Cosine Distance_{t_{k-1}}^{ \gamma }$$

The superscript and subscript represent the author and submission time points of the drafts, respectively.

A negative value of $\Delta Cosine{Distance}_{{t}_{k}}^{\theta }$ indicates that the revision made the focused version closer to other stories in the SCP-Wiki community. Conversely, a positive value indicates that the revision made the focused version further from other stories in the SCP-Wiki community.

**Figure 4:**
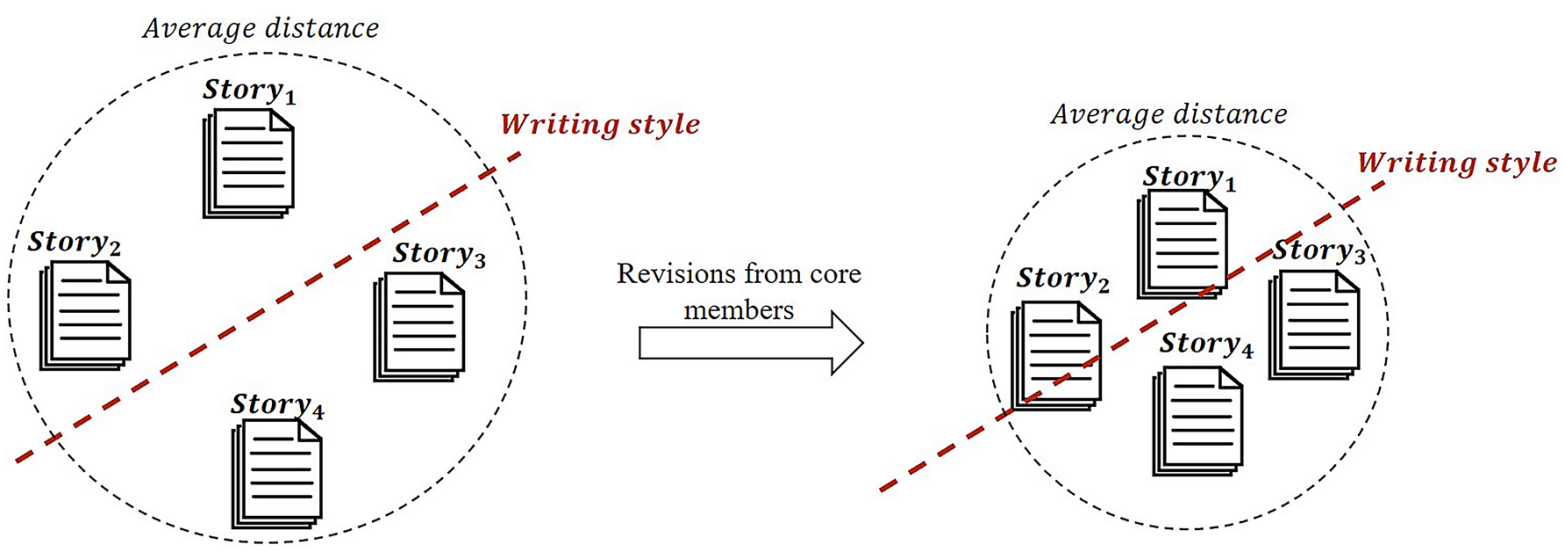
The illustration of the integration role of core members. The decreases in the average cosine distance among the versions of stories can be explained as an integration of the writing style among SCP-stories: with revisions by the core members, different SCP-stories began to share the same writing style; as a result, the average distance between them became smaller. The icon of stories in this figure was drawn by the first author using *Microsoft Paint* (version: *Windows 10 20H2*; URL: https://support.microsoft.com/en-us/windows/get-microsoft-paint-a6b9578c-ed1c-5b09-0699-4ed8115f9aa9).

Using a linear regression model (i.e., OLS), we examined the relationship between the changes in the average cosine distances and the values of the core-periphery metrics of the revised manuscript. In the regression, we controlled for the following values: 1) the average cosine distance of the *k* − 1 *times* version (i.e., $Cosine{Distance}_{{t}_{k-1}}^{ \gamma }$); 2) the number of content revisions; 3) the time point of the publication of the focal version; 4) the number of days from the revision’s first participation (i.e., submission or revision) until now (which also represented the number of days for the revision); 5) the revisor’s number of previous participations; and 6) the SCP story ID of the corresponding revision (as dummy variables that only affected the intercept). Variables 4) and 5), which indicated the previous experience of the revisors, were added because previous studies^26–28^ found that participants with more experience tended to present a more conservative attitude toward contents with larger average cosine distances. All regression results (in Table 4) showed significant negative correlations between the values of the core-periphery metrics of the revision and the change in the average cosine distance (for degree: *coefficient* =  − 0.01, *p-value* < 0.01; for k-core: *coefficient* =  − 0.03, *p-value* < 0.01; for eigenvector centrality: *coefficient* =  − 0.02, *p-value* < 0.01). Moreover, in Table S6 in ‘Supplementary Information’, we show that the results using the betweenness centrality are consistent with the results in Table 4. Based on the results of previous studies^45–47^, we consider that the decrease in the average cosine distance among the versions of stories can be explained as reflecting the integration of the writing style among SCP-stories (see the illustration in Fig. 4), through revisions by the core members, different SCP-stories began to share the same writing style; as a result, the average distance between them became smaller, and through this integration of writing style, the quality of the final version of the SCP-stories was also improved.

**Table 4:** Results of the regression on the change of originality for SCP-Wiki data.

Dependent variable: change of cosine distance among SCP-Wiki drafts					
Core-periphery metric: degree		Core-periphery metric: k-core		Core-periphery metric: eigenvector centrality	
Variable	Coefficient	Variable	Coefficient	Variable	Coefficient
Degree of revisor in logarithm scale	− 0.01** (0.005)	K− core of revisor in logarithm scale	− 0.03*** (0.004)	Eigen− vector centrality of revisor in logarithm scale	− 0.02*** (0.006)
Average cosine distance of the previous version	− 1.88*** (0.01)	Average cosine distance of the previous version	− 1.88*** (0.01)	Average cosine distance of the previous version	− 1.88*** (0.01)
Number of content revisions	0.05*** (0.008)	Number of content revisions in logarithm scale	0.05*** (0.008)	Number of content revisions in logarithm scale	0.05*** (0.008)
Time point of publication	− 0.02*** (0.008)	Time point of publication	− 0.03*** (0.008)	Time point of publication	− 0.02*** (0.008)
Days the revisor was observed	− 0.02 (0.004)	Days the revisor was observed	− 0.02*** (0.004)	Days the revisor was observed	− 0.01*** (0.004)
The revisor’s number of previous participations	0.002 (0.005)	The revisor’s number of previous participations	0.001 (0.004)	The revisor’s number of previous participations	0.007 (0.005)
Average constant^#^	− 0.093	Average constant^#^	− 0.092	Average constant^#^	− 0.092
*R*^2^	0.440	*R*^2^	0.441	*R*^2^	0.440
Observations: 43,693					

*p < 0.1; **p < 0.05; ***p < 0.01; Standard Error is shown in ( ); all coefficients in the table were estimated based on the standardized variables. Thus, the sizes of the coefficients are comparable. Since the regression estimated a different constant for each SCP-story, there were 4653 different constants. For simplification, we report the average constant here.

## Discussion

Based on the above results, this study has identified the different roles of different participants in co-creation: peripheral members create and core members integrate. Specifically, the peripheral members generated most of the initial content submissions in the co-creation process. Based on these initial contents, core members provided revisions and integrations, which improved the quality of the final outcomes in the co-creation.

Based on these results, our study makes two main contributions. First, in previous studies^8–18^, cooperation behaviours were always considered to be homogeneous, ignoring the ways in which different participants might play different roles in co-creation. In this regard, some previous studies^9,10^ have discussed cases in which participants had unequal roles (i.e., different abilities) that affected the outcomes in co-creation. However, even in these studies, cooperation behaviour was considered homogeneous (e.g., the cooperation strategy in PD). The unequal roles of participants were caused by their choices’ different influences (e.g., how strong the neighbours’ strategy choices can be affected by the focal participant in the interactions in the PD). In this study, we discussed the cooperation pattern among participants based on a co-creation model that considered different roles in co-creation (i.e., the originator *vs.* the revisor). In this regard, our research provides a deeper understanding of cooperation patterns in creative activities. In addition, our results can also contribute to co-creation practices in the real world. As explained above, we found that to boost the quantity of creative outcomes in co-creation, the key factor was the participation of peripheral members. In contrast, to ensure the quality of creative outcomes in co-creation, the key factor was the participation of core members. In this regard, our results not only explained how cooperation occurred in creative activities but also pointed out how co-creation can be effectively organised. By organising a team comprising participants with diverse network positions (i.e., both core and peripheral members), the co-creation team is expected to provide a large quantity of high-quality creative outcomes.

In addition to the above implications, we also provide a further interpretation of the relationship between participants’ different roles and their different network positions. The different roles of core and peripheral members in co-creation can be attributed to the different information they possess. In this respect, on the one hand, peripheral members often have more chances to obtain divergent (i.e., unrelated) information than core members through the network^26–28^. On the other hand, core members often have a deeper understanding of the criteria of the community (e.g., a consensus on what content should be published in the community) than peripheral members^26–28^. Following the results of previous studies, we consider that initial content submissions in co-creations require more divergent information. As a result, initial content submissions were mainly made by peripheral members. By contrast, revisions in the co-creation are mainly based on the criteria of the community. As a result, revisions were mainly made by core members.

Finally, it is worth noting that although the cooperation patterns among participants were the same in our three datasets, there were still some differences among the results obtained from the different datasets. Importantly, the peripheral members in the GitHub dataset had a significantly larger likelihood of engaging in initial content generation compared to the peripheral members in the other two datasets (to see details, comparing the starts of the blue lines in the same column in Fig. 2). We consider that this difference may be caused by the individual features of peripheral members. Previous research^29–31,48^ implied that compared to SCP-Wiki members and Idea Storm members, GitHub members tended to have more professional knowledge, although the types of their professional knowledge differed: SCP-Wiki members possessed knowledge on story writing, Idea Storm members were proficient in product design, and GitHub members possessed knowledge about software development. Therefore, the differences between the results obtained for the three datasets implies that the professional knowledge of the corresponding creative activities may enhance the peripheral members’ tendency to generate initial content. In this respect, although this research emphasises the relationship between network positions and the roles of participants, individual features (e.g., the amount of professional knowledge) should also be considered in further discussions of cooperation patterns in co-creation. By using datasets that record more comprehensive information from the participants, this issue may be investigated in more detail.

## Materials and methods

### Description of three datasets: SCP-Wiki, GitHub, and Idea Storm

In this study, we obtained data from SCP-Wiki (http://www.scp-wiki.net/), GitHub (https://github.com/), and Dell’s Idea Storm (http://www.ideastorm.com/) to investigate the different roles of participants in co-creation. SCP-Wiki is a website that participants use to submit and discuss their original science fiction stories^29^. Its data records the author, content, and time point for each SCP-story submission and revision. GitHub is one of the largest software development platforms in the world^30^. GitHub data records the author and the time point of each source code upload and revision. Idea Storm is a website designed by Dell to collect interesting ideas from users^31^. The Idea Storm data records the author and the time point for each idea submission and discussion.

Using the APIs provided by these three websites, we gathered a) 92,477 drafts (i.e., different revised versions) of 4653 SCP-stories submitted by 3405 participants in the SCP-Wiki from January 2009 to December 2018; b) 99,230 code repertories from January 2010 to April 2020, which were programmed in *Python* and received at least one *pull request* (that is, a revision from other participants; see details in the next section); and c) 837 ideas that had at least one *comment* from other participants (see details in the next section) from 6333 members from February 2007 to October 2018.

### Construction of cooperation networks

We constructed cooperation networks for our three datasets based on the cooperation relationships between participants. In general, all three networks are undirected weighted networks. The connections (i.e., edges) indicate that the two participants cooperate on the same content, and the weights of the connections indicate the times of cooperation between the two participants. Specifically, in SCP-Wiki, if two participants had worked on the same SCP story either as an originator or a revisor, they were connected in the cooperation network. In GitHub, the owners of the code repertories (i.e., originators) and the participants who submitted the *pull request* for the code repertories (i.e., the revisions) were connected. A pull request is a revision sent by a revisor to request that the owner of a code repertory updates the source code. In Idea Storm, the originators of the ideas and the participants who submitted *comments* on the ideas (i.e., the revisors) were connected. The comment is a function in Idea Storm through which members discuss how to refine each other’s ideas. In this way, we constructed three cooperation networks for the three datasets.

In the ‘S3’ section in ‘Supplementary Information’, we provide the topology features (i.e., the degree distributions and the rich-club coefficients) of the three cooperation networks. We found that the three networks were not scale-free, although their degree distributions followed the power-law distribution. Moreover, based on the rich-club coefficients, there were several subgroups in the cooperation networks of the SCP-Wiki and GitHub communities; in contrast, in the Idea Storm community, these subgroups did not exist.

### Computation of core-periphery metrics and their normalization

Previous research^13–20,32–40^ has found two ways to identify the core members in networks: 1) based on the connections among nodes (i.e., participants), and 2) based on the information flow among nodes. In this study, we use both of these methods.

Based on the connections among nodes, we employed two indicators: *degree* and *k-core*. The value of the degree indicates the number of other nodes connected to the focal node. Therefore, a larger degree value indicates that the focal node has more connections with other nodes in the network. Additionally, previous research^13–18^ found that core members not only have a high number of connections but also a high density of connections around them. In other words, core members are always in the cohesive subgroups of the network. To consider both the number of connections of the focal node and the cohesiveness of its subgroup, we also employed *a k-core* (also called *k-plex*)^35–38^. A node with the value *k*, according to the *k-core*, is in a subgraph in which each node is connected to *k* other nodes in this subgroup. In other words, a larger *k-core* value indicates that the focal node has more connections with other nodes and is in a more cohesive subgroup.

We also used *eigenvector centrality* as an indicator based on the information flow among the nodes. The eigenvalue centrality identifies the information hubs of the network^32–34,38–40^. Intuitively, the computation of eigenvector centrality can be considered as a voting process in the network; at first, each node in the network is assigned the same number of votes. Then, in each iteration of voting, the focal node deploys its votes to its neighbours (i.e., other nodes have connections with the focal node) based on the weights of the connections. Meanwhile, the focal node also receives votes from its neighbours and uses these votes to vote in the next iteration. After a sufficiently large number of iterations, each node’s votes will converge, and this number is taken as the given node’s eigenvector centrality^27^. In mathematical terms, the number of votes will converge to the components of the first eigenvector (i.e., the eigenvector with the largest eigenvalue) of the adjacency matrix of the network (i.e., a matrix using zero and positive numbers, respectively, to represent whether a connection exists between two nodes)^34^. Therefore, a larger eigenvector centrality indicates that the focal node receives a larger number of votes from its neighbours. If we consider the voting process as an information diffusion process, nodes with a large eigenvector centrality can be considered information hubs in the network.

In summary, we used degree, k-core, and eigenvector centrality to identify the core members of the networks. For all three indicators, a larger value of the metrics indicates that the focal node is closer to the core of the network. Additionally, in this study, we compared the values of the above three metrics between different time points to identify the core members of the networks at different times. However, because the networks also changed with time, the values of the metrics at different time points were not directly comparable. To solve this problem, we normalised our metrics over time to ensure that their values indicated the same core-periphery position, even at different time points. For degree and k-core, we used the number of connections in the whole network at a certain time point to normalise their values (i.e., $\widehat{\mathrm{X}}=\frac{\mathrm{X}}{|\mathrm{E}|}$, where $\widehat{\mathrm{X}}$ is the normalised value of the metric and $|\mathrm{E}|$ is the number of connections in the whole network at a certain time point). We normalised eigenvector centrality by the norm of the first eigenvector at a certain time point. In this way, regardless of the difference in time, a large value of one of our metrics indicates that the focal node was closer to the core of the network.

Finally, as mentioned in the “Results” section, in the ‘S4’ section in ‘Supplementary Information’, we also used betweenness centrality to measure the core-periphery positions of members in the networks^41,42^. This indicator measures how many shortcuts (i.e., the shortest path between a pair of nodes; see details in the ‘S4’ section in ‘Supplementary Information’) in the network pass the focal node^41,42^. Thus, a node with a large betweenness centrality can be considered as an information hub^41^, namely, a core member. Note that, in the specific analyses, we used an approximation algorithm rather than its original definition to compute betweenness centrality (see details in the ‘S4’ section in ‘Supplementary Information’), because its computation is far more time-consuming than the other three core-periphery metrics in the manuscript^41^. As the ‘S4’ section in ‘Supplementary Information’ shows, the results based on betweenness centrality were all consistent with the results obtained on the basis of other metrics.

### Quality metrics of the content and the regression models

We used three different metrics to measure the quality of content in our datasets. In SCP-Wiki, we used the *rate* to measure the quality of the story^29^. The rate is a participant-generated indicator. Every registered participant in the SCP-Wiki can either vote ‘1’ for an SCP-story, which indicates that the content of the story is good, or vote ‘− 1’ for the story, which indicates that the content is not good. The final value of *the rate* for an SCP-story is the sum of all ‘1’ and ‘− 1’ ratings. For instance, 201 participants voted for scp − 001 (i.e., the first story in the SCP-website); 191 of them voted ‘1’ and 10 of them voted ‘− 1’. In the present case, the scp-001 rating was 181. In the GitHub data, we employed the number of *stars* for each code repertory to measure its quality^30^. Stars simply indicated how many registered participants in GitHub thought that the source code in the repertory was good. Finally, in Idea Storm, according to previous research^31,48^, we used *idea status* to measure idea quality, a five-class metric generated by a specialist team at Dell that presents an evaluation of the submitted ideas. It includes ‘Submitted’, ‘Archived’, ‘Acknowledged’, ‘Partly Implemented’, and ‘Implemented’. According to previous research^31,48^, ideas with a ‘Partly Implemented’ or ‘Implemented’ status were defined as valuable ideas. Therefore, in Idea Storm, the quality of ideas is a binary variable with a value of 0 (i.e., *not valuable*) or 1 (i.e., *valuable*).

To examine the relationship between the originator’s value of the core-periphery metrics and the quality of the content, we conducted three regression models. For the SCP-Wiki and GitHub data, we used linear regression models (i.e., OLS). For the Idea Storm data, we used a binary logistic regression model because the quality of ideas was a binary variable. The regression formula is as follows:

For the SCP-Wiki data:

$$\begin{aligned} & Rate \,on \,logarithmic \,scale \\ &\quad={ \alpha }_{1}\,Core\, Periphery \,Metric \,of \,the \,originator \,on \,logarithmic \,scale\\ &\quad\quad+{\alpha }_{2}\,Average \,K{-}core \,of \,revisors \,on \,logarithmic \,scale\\ &\quad\quad+{\alpha }_{3}\,Number \,of \,content \,revisions \,on \,logarithmic \,scale\\ &\quad\quad+{\alpha }_{4}\,Time \,point \,of \,publication+{\alpha }_{5}\,Days \,being \,observed \,of \,the \,originator\\ &\quad\quad+{\alpha }_{6}\,The \,originator{\text{'}}s \,number \,of \,previous \,participations+{\alpha }_{0}\end{aligned}$$

For the GitHub data:

$$\begin{aligned} & Number \,of \,Stars \,on \,logarithmic \,scale\\ &\quad={ \alpha }_{1}\,Core \,Periphery \,Metric \,of \,the \,originator \,on \,logarithmic \,scale\\&\qquad+{\alpha }_{2}\,Average \,K{-}core \,of \,revisors \,on \,logarithmic \,scale\\&\qquad+{\alpha }_{3}\,Number \,of \,content \,revisions \,on \,logarithmic \,scale\\&\qquad+{\alpha }_{4}\,Time \,point \,of \,publication+{\alpha }_{5}\,Days \,being \,observed \,of \,the \,originator\\&\qquad+{\alpha }_{6}\,The \,originator{\text{'}}s \,number \,of \,previous \,participations +{\alpha }_{0}\end{aligned}$$

For the Idea Storm data,

$$\begin{aligned}& Odds \,Ratio\,(the \,idea \,is \,valuable \,or \,not)\\ &\quad=\Lambda ({ \alpha }_{1}\,Core \,Periphery \,Metric \,of \,the \,originator \,on \,logarithmic \,scale\\ &\quad\quad+{\alpha }_{2}\,Average \,K{-}core \,of \,revisors \,on \,logarithmic \,scale\\ &\quad\quad+{\alpha }_{3}\,Number \,of \,content \,revisions \,on \,logarithmic \,scale\\ &\quad\quad+{\alpha }_{4}\,Time \,point \,of \,publication+{\alpha }_{5}\,Days \,being \,observed \,of \,the \,originator\\ &\quad\quad+{\alpha }_{6}\,The \,originator{\text{'}}s \,number \,of \,previous \,participations +{\alpha }_{0})\end{aligned}$$

Here, $\Lambda \left(\overrightarrow{{\varvec{v}}}\right)$ is the logistic function, ${\left(1+{e}^{-\overrightarrow{{\varvec{v}}}}\right)}^{-1}$, and the ‘core periphery metric’ is the value of one of the three indicators—that is, degree, k-core, and eigenvector centrality—for the originator.

Since the quality of one type of content was affected both by the features of the originator and the features of the revisors^26–28^, in all models, we added 1) the average value of the core-periphery metrics of all corresponding revisions; 2) the number of content revisions; 3) the time point of the content publication; 4) the number of days from the originator’s first participation (i.e., submission or revision) until now, and 5) the originator’s number of previous participations as control variables. As explained in the “Results” section, control variables 4) and 5), which indicate the originator’s previous experience, were added because previous research^26–28^ indicated that contents from experienced participants (i.e., originators) in the communities (i.e., the websites) tend to be overestimated. In contrast, the content from inexperienced participants in the communities tended to be underestimated.

The statistics of all the variables in the three regressions can be found in ‘Supplementary Information’.

### Document-embedding and revision behaviour analysis in SCP-Wiki

We used SCP-Wiki data to examine how revision behaviours changed the content. SCP-Wiki provides a *history* function that allows users to see the previous drafts (i.e., previous versions) of stories and to compare the previous text with the revised text. To measure content changes, we used sentence embedding based on the latest representation model for natural language: BERT^43^ (hereafter referred to as *sentence-BERT*). Since BERT uses contextual word-embedding^44^, sentence-BERT can capture trivial semantic changes in the revisions^43^. According to a previous study^43^, this sentence embedding model outperformed other document-embedding models in text classification tasks. Additionally, the cosine distance between the embedding results of this model showed a strong correlation with the semantic relationship between the embedded texts^43–46^. As explained in the “Results” section, we first calculated the average cosine distance between the focal version and the versions of all SCP-stories. We then traced the change in average distances with each revision.

We constructed a multiple linear regression model (i.e., OLS) to examine the relationship between the values of the core-periphery metrics of the revised version and the change in the average distance of the focal version. It is worth noting that since not all revisions changed the text content of the story (e.g., some only added pictures, revised punctuation, or adjusted the display), we finally focused on the revisions that changed the text contents (in total, we analysed 43,693 revisions). The formula for the regression model is as follows:

$$\begin{aligned} & Change \,of \,average \,cosine \,distance \\ &\quad={ \alpha }_{1}\,Core \,Periphery \,Metric \,on \,logarithmic \,scale\\ &\quad\quad+{\alpha }_{2}\,Average \,Cosine \,Distance \,of \,the \,previous \,version\\ &\quad\quad+{\alpha }_{3}\,Number \,of \,content \,revisions \,on \,logarithmic \,scale\\ &\quad\quad+{\alpha }_{4}\,Time \,point \,of \,publication+{\alpha }_{5}\,Days \,being \,observed \,of \,the \,revisor\\ &\quad\quad+{\alpha }_{6}\,The \,revisor{\text{'}}s \,number \,of \,previous \,participations+{\alpha }_{0}|ID \,of \,SCP \,Story\end{aligned}$$

Here, the ‘core periphery metric’ is the value of one of the three indicators; that is, degree, k-core, and eigenvector centrality, for the focal node.

In the regression, we controlled for the following values: 1) the average cosine distance of the *k* − 1-*times* version (i.e., $Cosine{Distance}_{{t}_{k-1}}^{ \gamma }$), 2) the number of content revisions, 3) the time point of the story publication, 4) the number of days from the revisor’s first participation (i.e., submission or revision) until now (which also represented the days the revision was observed); 5) the revisor’s number of previous participations; and 6) the SCP story ID of the corresponding revision (as dummy variables that only affected the intercept). Control variables 4) and 5), which indicate the previous experience of the revision, were added because previous studies^26–28^ suggested that experienced participants in communities (i.e., the website) are largely constrained by their previous experience and tend to be more conservative toward versions with a large average distance. Therefore, experienced participants tend to reduce their average distance in the focal versions^26–28^.

Additionally, the ${\alpha }_{0}|ID\:of\:SCP\:Story$ in the formula indicated that the unique ID of each SCP-story, which was added as a fixed effect in the model, only affected the constants^49^. Thus, the model estimated 4653 different constants for drafts with 4653 different stories.

The statistics for each variable in the model are shown in ‘Supplementary Information’.

## Supplementary Information

Supplementary Information.

## Data Availability

The R-code during the current study and the three datasets analysed during the current study (including data for making Figures) are available in the Mendeley Data: http://dx.doi.org/10.17632/t8nxnf472r.1.
