# Supplementary material for: Cooperation patterns of members in networks during co-creation
Source: Sci Rep. 2021 Jun 8;11:11588. doi: 10.1038/s41598-021-90974-1 (PMC8187372; doi:10.1038/s41598-021-90974-1)
Supplement: Supplementary file 1 — Supplementary Information. [file 41598_2021_90974_MOESM1_ESM.pdf]

Supplementary Information for

Cooperation patterns of members in networks during co-creation

Authors: Kunhao Yang[1]*, Itsuki Fujisaki[1,2], Kazuhiro Ueda[1]*

[1] Graduate School of Arts and Sciences, The University of Tokyo, Tokyo, Japan (〒153-8902).

[2] Research Fellowship for Young Scientists (DC2), Japan Society for the Promotion of Science (JSPS), Tokyo, Japan (〒102-0083).

* Kunhao Yang, Kazuhiro Ueda

Email: yangkunhao@g.ecc.u-tokyo.ac.jp; ueda@gregorio.c.u-tokyo.ac.jp

This PDF file includes:

Supplementary text

Figures S1 to S11

Tables S1 to S6

Supplementary Information Text

S1. Statistical information about the regression models of the quality of content

Table S1 shows the statistics of all variables in the three regression models for the quality of content (Tables 1–3 in the manuscript). The distributions of each variable and the correlations between them are shown in Figs. S1–S3.

-----Table S1 about here-----

-----Fig. S1 about here-----

-----Fig. S2 about here-----

-----Fig. S3 about here-----

S2. Statistical information about the regression model of revision behaviours in SCP-Wiki

The statistical information for each variable in the regression model of revision behaviours in SCP-Wiki (in Table 4 in the manuscript) is shown in Table S2. The distributions of each variable and the correlations between them are shown in Fig. S4.

-----Table S2 about here-----

-----Fig. S4 about here-----

S3. Topology features of the three cooperation networks

The degree distributions and rich-club coefficients of the three cooperation networks are shown in Fig. S5. The panels in the first column of Fig. S5 show the degree distributions of the three networks. We found that the node degrees in the three networks followed a *power-law* distribution. Namely, the possibility of nodes with degree k is related to the degree k in a power law ($P_k \sim C \cdot\ k^{-\gamma}\ where\ C\ and\ \gamma\ are\ two\ constants$).[1] To interpret these distributions, the power-law distribution indicates that most of the nodes in the three networks had very small degrees, and a small number of nodes had very large degrees. Then, we estimated the values of γ by OLS regressions to examine whether the three networks were scale-free.[1] We found that the three networks' γs were larger than one but smaller than two (γ =1.26 in the SCP-Wiki data; γ =1.64 in the SCP-Wiki data; γ =1.21 in the Idea Storm data). These results indicate that the three networks are not scale-free. This could be attributed to the fact that all members in the three communities were given equal opportunities to cooperate: both the newcomer and the experienced members had a chance to cooperate with other members; thus, the frequency of nodes with the corresponding degree does not decrease significantly when the value of the degree increases.

The panels in the second column of Fig. S5 shows the rich-club coefficients of the three networks, which reflect the extent to which well-connected nodes (i.e., nodes with high degrees) connect to each other in the three networks. The specific computation of the rich-club coefficient is as follows[2]:

$$\phi(k) = \frac{2E_{>k}}{N_{>k}(N_{>k} - 1)}$$

where $E_{>k}$ is the number of edges between the nodes with degrees greater than or equal to k, and $N_{>k}$ is the number of nodes with degrees greater than or equal to k. This indicator measures the number of connections among nodes with degrees at least k, normalised by the number of connections that exist between these nodes at most. An interesting point in Fig. S5 shows that in SCP-Wiki data and GitHub data, when the value of k was larger than a certain value (the logarithm of k was larger than 6 in SCP-Wiki data and the logarithm of k was larger than 8 in the GitHub data), the corresponding rich-club coefficients became zero; however, in Idea Storm data, regardless of the value of k, the corresponding values of rich-club coefficients kept increasing. These results imply that there exist several subgroups in the cooperation networks of the SCP-Wiki and GitHub communities. These subgroups have a centre node with a very large degree as well as some member nodes with small degrees around the centre node. As a result, the nodes with large degrees (i.e., the centre nodes) are only connected to other nodes with smaller degrees (i.e., the member nodes). In contrast, in the cooperation network of the Idea Storm community, these subgroups did not exist. As a result, all nodes with large degrees are connected to each other.

S4. Results based on the betweenness centrality

As explained in the manuscript, in addition to degree, k-core, and eigenvector centrality, we also employed the betweenness centrality to measure the core-periphery positions of nodes in the network. In this section, we first explain the definition and computation of betweenness centrality. We then report the results based on the betweenness centrality in detail.

Simply speaking, the betweenness centrality measures the number of shortest paths in the network passing through the focal node.[3,4] The shortest path between two nodes is defined as the path connecting these two nodes by passing the fewest nodes. Its computational formula is as follows:

$$g(v) = \sum_{s \neq v \neq t} \frac{\sigma_{st}(v)}{\sigma_{st}}$$

where σ_{st} is the total number of shortest paths from node *s* to node *t*, and $\sigma_{st}(v)$ is the number of those paths that pass through node v. Based on this computation, a node with a large betweenness centrality can be considered to be a node bridging a large number of nodes through the short paths among these nodes. Therefore, the focal node can be considered as an information hub,[3] namely, a core member, in the network. Note that in the specific computation, we computed the betweenness centrality using an approximation algorithm with a cut-off value of 3. In other words, when computing node *v*'s betweenness centrality, we only considered the shortest paths between nodes whose distance to *v* was equal to or less than 3. We used this approximation algorithm because the computation of betweenness centrality is far more time-consuming than the other three core-periphery metrics in the manuscript.[3,4]

After we obtained the value of each node's betweenness centrality, we normalised its value over time to ensure that the value indicated the same core-periphery position across different time points. In the normalisation, we divided the values of betweenness centrality by $\frac{(N-1)(N-2)}{2}$ (i.e., $g(v)^{new} = \frac{2g(v)}{(N-1)(N-2)}$). *N* is the number of nodes whose distance to *v* is equal to or less than 3 at time point *t*. In this manner, regardless of the

difference in time, a large value of betweenness centrality indicates that the focal node is closer to the core of the network.

Based on the normalised values of the betweenness centrality, we replicated the same analyses based on the other three metrics in the manuscript. Because many previous studies have pointed out that although betweenness centrality is a very important metric for detecting subgroups in a network (i.e., community detection application)[4], it is not as effective as eigenvector centrality[5-7] and k-core[5] for identifying the specific core members (i.e., nodes) in networks, the results based on the betweenness centrality are only shown in this section.

In summary, the results based on betweenness centrality were consistent with the results in the manuscript. The results shown in Fig. S6 are the same as the results in Fig. 2 in the manuscript: 1) the red lines in Fig. S6 show that in all three datasets (i.e., SCP-Wiki, GitHub, and Idea Storm data), participants with smaller values of betweenness centrality (i.e., peripheral members) submitted a larger proportion of initial content, while participants with larger values of betweenness centrality (i.e., core members) contributed less initial content; 2) the blue lines in Fig. S6 show that in all three datasets, the participants with a smaller value of between centrality at time point t had a significantly larger likelihood of submitting initial contents at time point t+1; 3) the insets in Fig. S6 show that even after controlling for the time factor, the participants with smaller values of betweenness centrality (i.e., peripheral members) still had a significantly larger possibility of submitting initial contents at the next time point than those with larger values of betweenness centrality (i.e., core members).

The results shown in Fig. S7 and Tables S3-5 are also consistent with the results in Fig. 3 and Tables 1-3 in the manuscript. In particular, the regression results (the statistical information of the variables in the regressions and the correlations among them are shown in Table S1 and Figs. S8-10) in Tables S3-5 show that by controlling all the related factors, the values of the core-periphery metrics of the originators did not have a significant positive effect on content quality (i.e., the betweenness centrality did not have significant positive coefficients in any of the regression models; see the specific coefficients in the fourth row of Tables S3-5). More importantly, the average value of the betweenness centrality of the revisions had a significant positive effect on the final outcome quality.

Finally, the results in Table S6 are consistent with the results in Table 4. The regression results (the statistical information of the variables in the regressions and the correlations among them are shown in Table S2 and Fig. S11) showed a significant negative coefficient (*coefficient* = -0.018, *p-value* = 0.007) between the values of the betweenness centrality of the revised version and the change in the average cosine distance.

In summary, the results based on betweenness centrality supported the conclusion in the manuscript: in co-creations, the peripheral members generated most of the initial content submissions. Then, based on these initial contents, core members provide their revisions and integrations, which improve the quality of the final co-created outcomes.

References

1. Barabási, A. L. & Bonabeau, E. Scale-free networks. *Sci. Am.* **288**, 60-69 (2003).
2. Colizza, V., Flammini, A., Serrano, M. A. & Vespignani, A. Detecting rich-club ordering in complex networks. *Nature Phys.* **2**, 110-115 (2006).
3. Brandes, U. A faster algorithm for betweenness centrality. *J. Math. Sociol.* **25**, 163-177 (2001).
4. Arasteh, M. & Alizadeh, S. A fast divisive community detection algorithm based on edge degree betweenness centrality. *Appl. Intell.* **49**, 689-702 (2019).
5. Della Rossa, F., Dercole, F. & Piccardi, C. Profiling core-periphery network structure by random walkers. *Sci. Rep.* **3**, 1-8 (2013)
6. da Silva, M. R., Hongwu Ma & An-Ping Zeng Centrality, network capacity, and modularity as parameters to analyze the core-periphery structure in metabolic networks. *Proc. IEEE* **96**, 1411-1420 (2008)
7. Borgatti, S. P. & Everett, M. G. Models of core/periphery structures. *Soc. Netw.* **21**, 375-395 (2000).

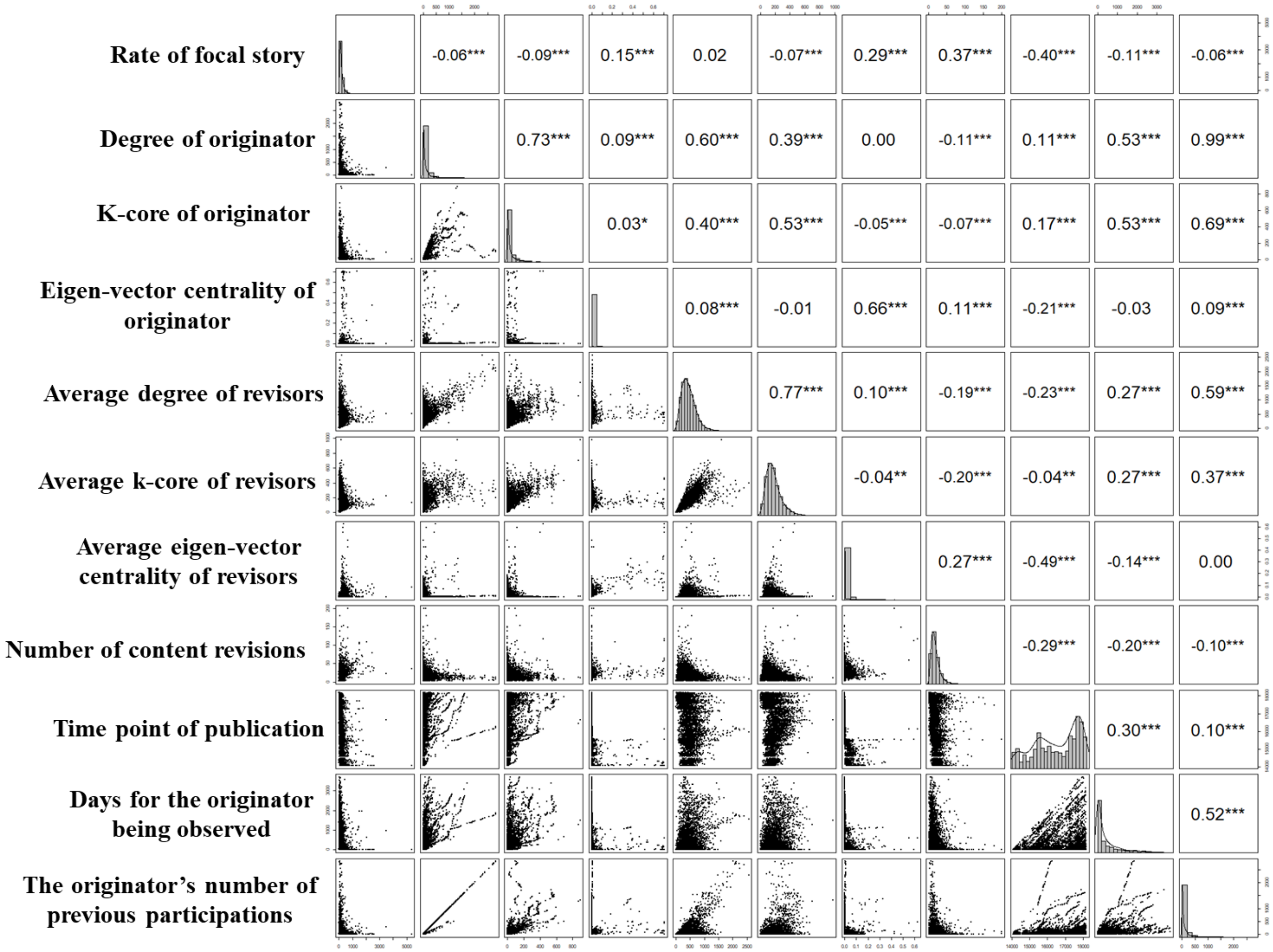

Fig. S1. Distribution, scatter plots, and correlations of variables in the regression model of SCP-Wiki content quality; the names of all variables are in the left of the figure. In this figure, the diagonal shows histograms with the density curves of each variable in the regression model of content quality in SCP-Wiki. Graphs in the lower triangle show scatter plots between each pair of variables. The upper triangle shows significant correlations between each pair of variables. One asterisk refers to a *p*-value smaller than 0.1, two asterisks refer to a *p*-value smaller than 0.05, and three asterisks refer to a *p*-value smaller than 0.01.

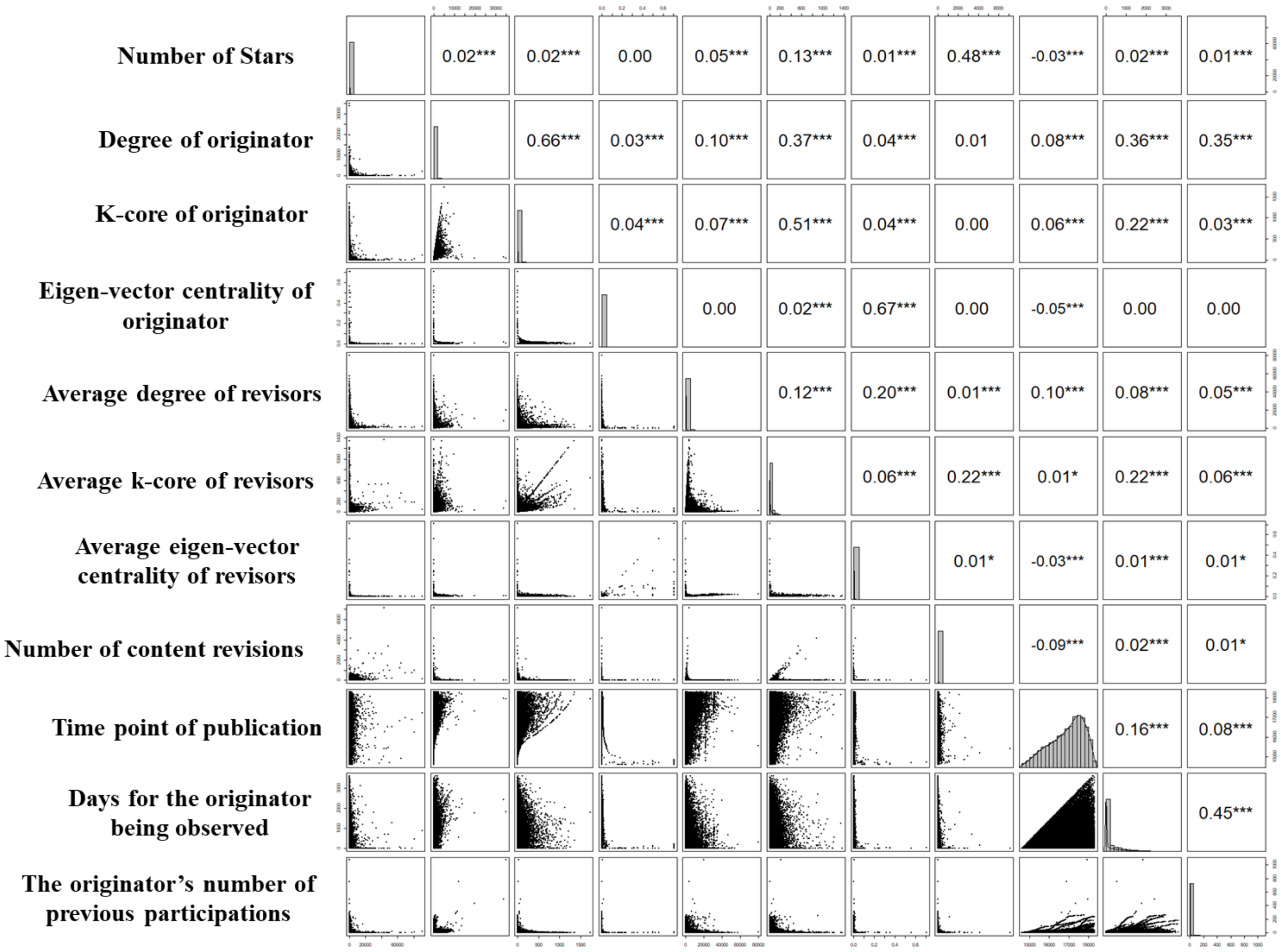

Fig. S2 Distribution, scatter plots, and correlations of variables in the regression model of GitHub content quality; the names of all variables are in the left of the figure. In this figure, the diagonal shows histograms with the density curves of each variable in the regression model of content quality in GitHub. Graphs in the lower triangle show scatter plots between each pair of variables. The upper triangle shows significant correlations between each pair of variables. One asterisk refers to a *p*-value smaller than 0.1, two asterisks refer to a *p*-value smaller than 0.05, and three asterisks refer to a *p*-value smaller than 0.01.

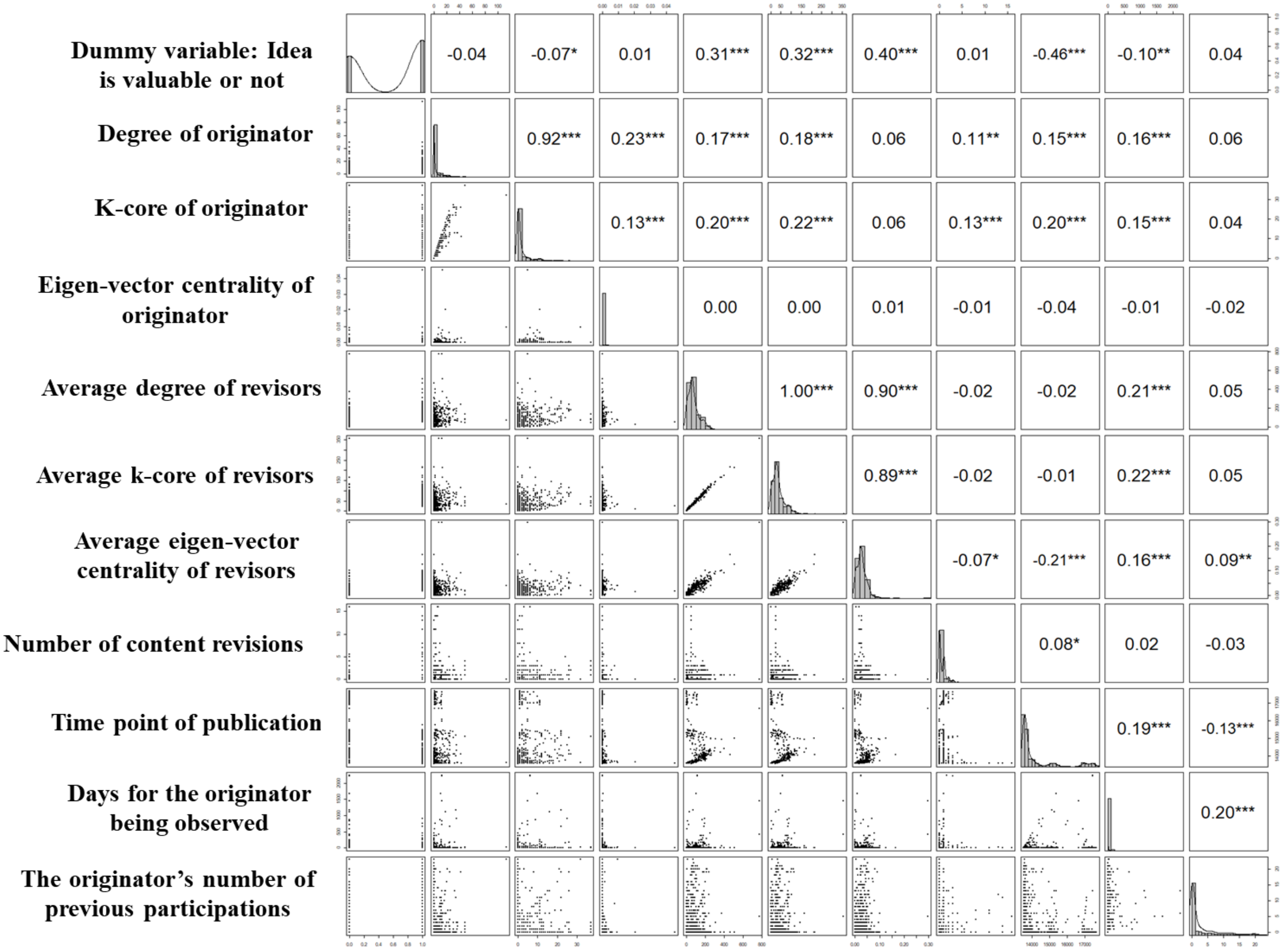

Fig. S3. Distribution, scatter plots, and correlations of all variables in the regression model of Idea Storm content quality; the names of all variables are in the left of the figure. Note that the names of the variables are abridged for display in the figure. In this figure, the diagonal shows histograms with the density curves of each variable in the regression model of content quality in Idea Storm. Graphs in the lower triangle show scatter plots between each pair of variables. The upper triangle shows significant correlations between each pair of variables. One asterisk refers to a *p*-value smaller than 0.1, two asterisks refer to a *p*-value smaller than 0.05, and three asterisks refer to a *p*-value smaller than 0.01.

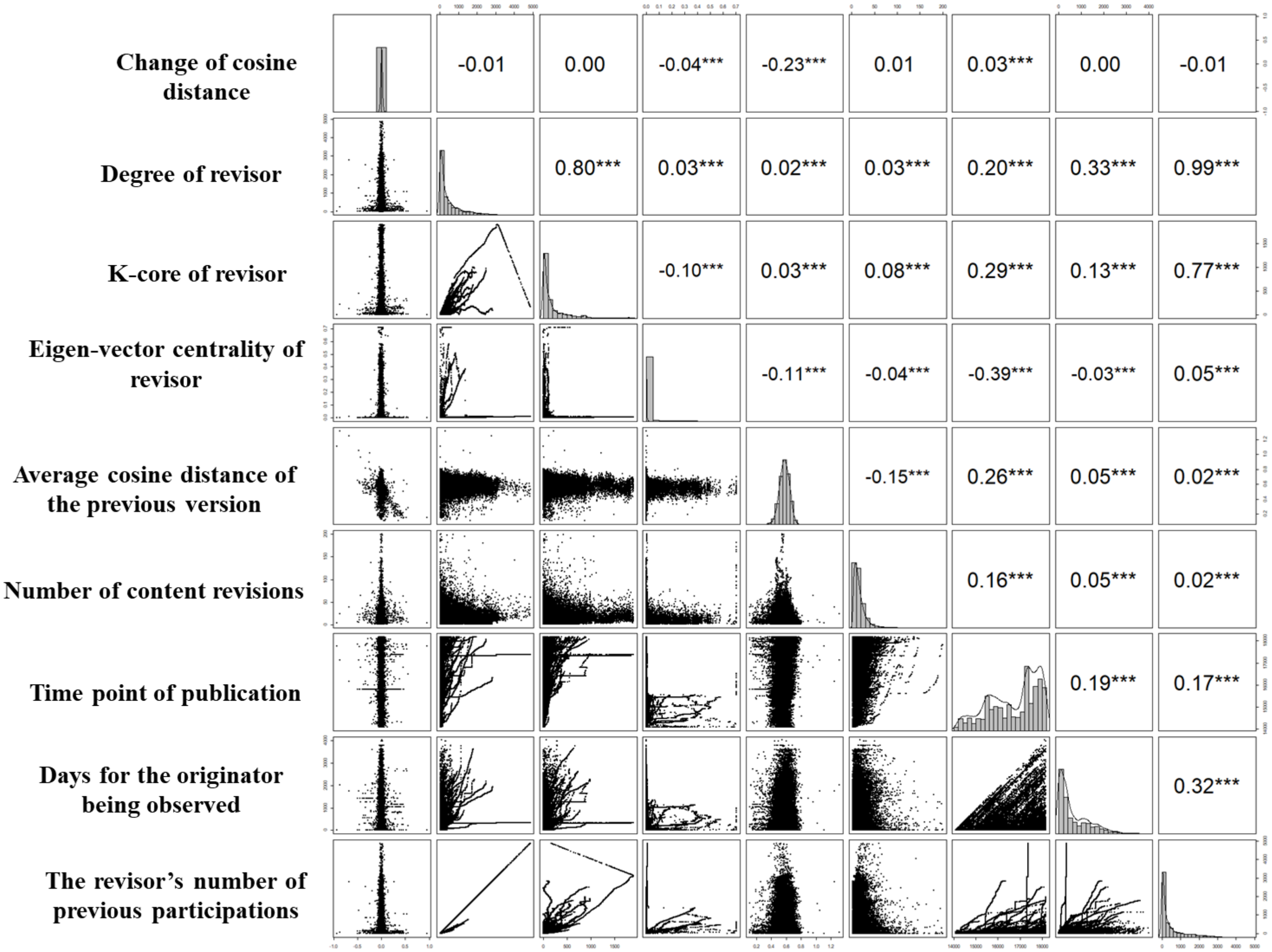

Fig. S4. Distribution, scatter plots, and correlations of variables in the regression model of originality change; the names of all variables are in the left of the figure. In this figure, the diagonal shows histograms with the density curves of each variable in the regression model of originality change. Graphs in the lower triangle show scatter plots between each pair of variables. The upper triangle shows significant correlations between each pair of variables. One asterisk refers to a *p*-value smaller than 0.1, two asterisks refer to a *p*-value smaller than 0.05, and three asterisks refer to a *p*-value smaller than 0.01.

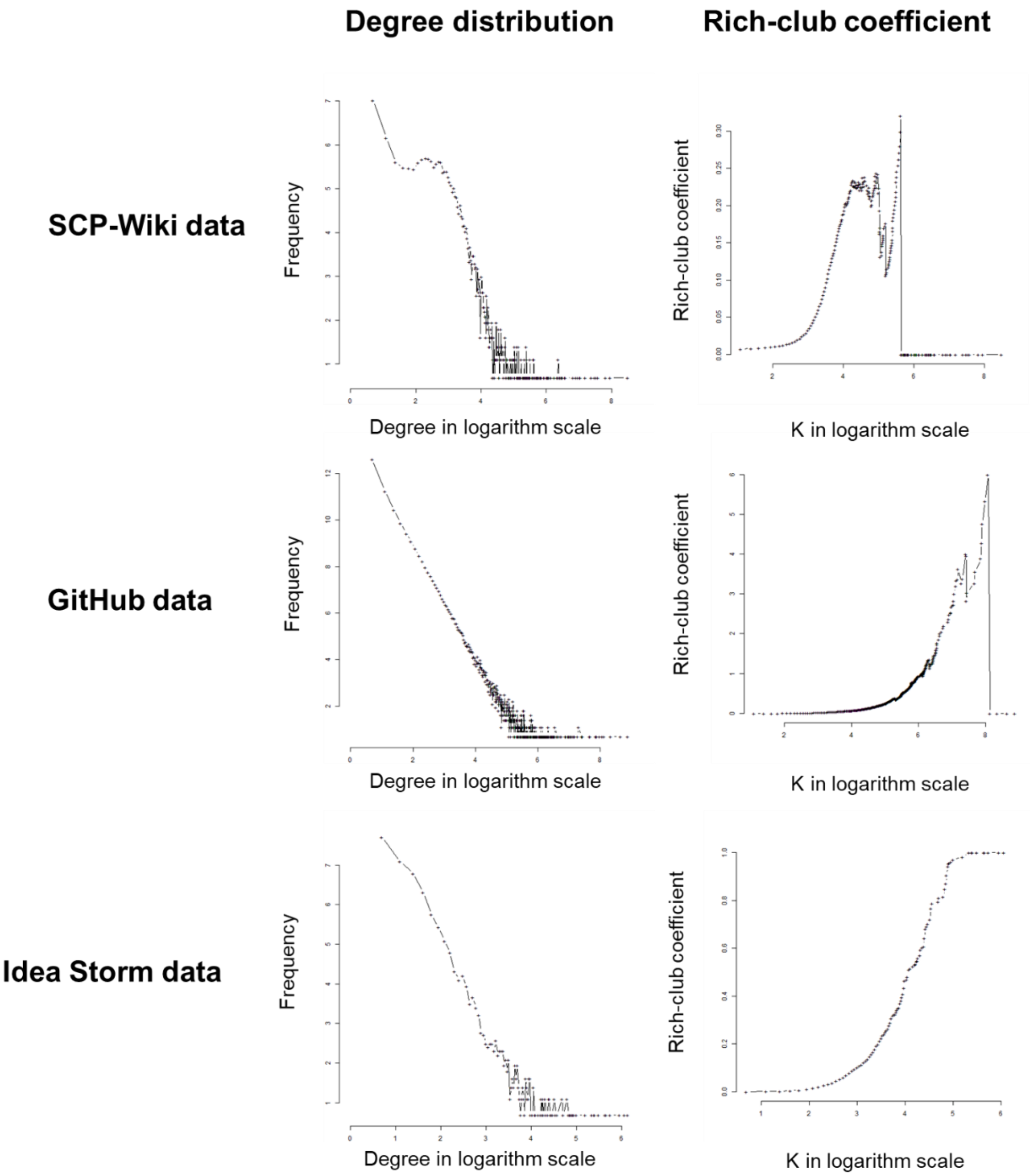

Fig. S5 Degree distributions and rich-club coefficients of the three datasets. The panels in the first column show the degree distributions of the cooperation network in SCP-Wiki data, GitHub data and Idea Storm data, respectively. The x-axes in these panels represent the values of node's degree in logarithm; the y-axes represent the node's frequency in logarithm scale. The panels in the second column show the rich-club coefficients of the cooperation network in SCP-Wiki data, GitHub data and Idea Storm data, respectively. The x-axes in these panels represent the values of *k* in the computation formula of the rich-club coefficient. The y-axes represent the corresponding rich-club coefficients.

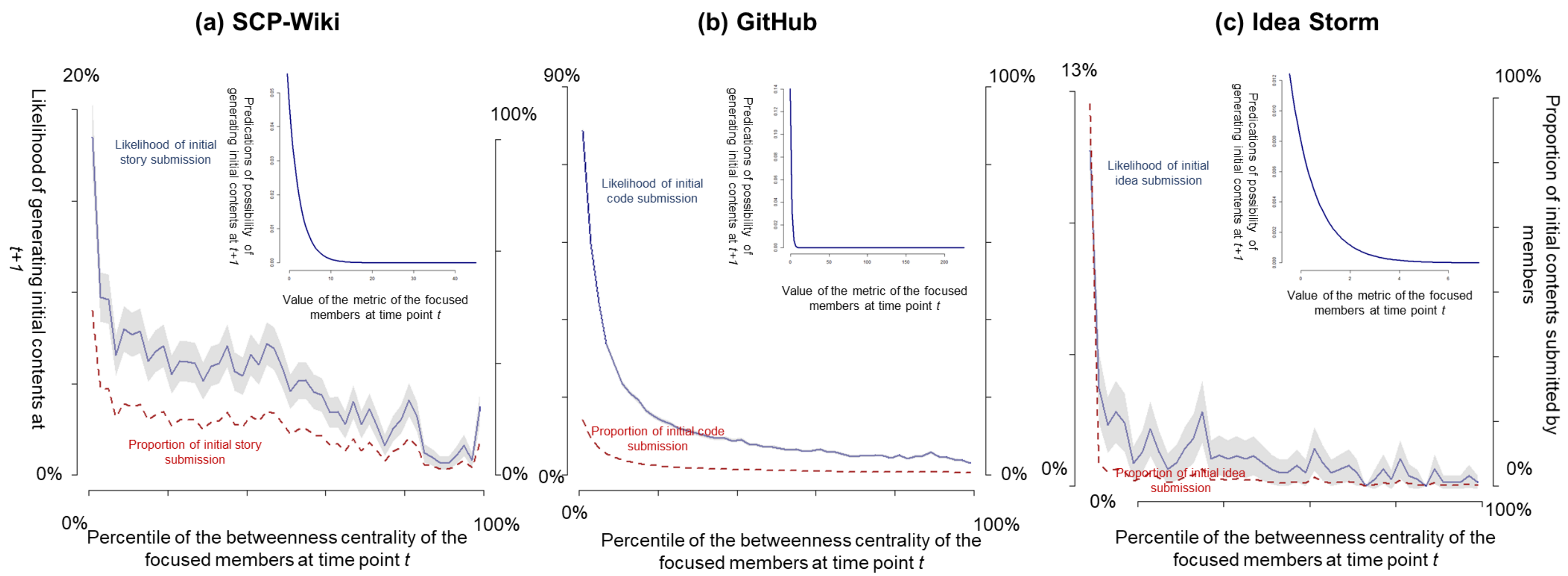

Fig. S6 Illustration of the relationship between the betweenness centrality and the initial content submissions; In Fig. S6, the relationships between the different values of the betweenness centrality and the initial content submission in (a) SCP-Wiki, (b) GitHub, and (c) Idea Storm are shown for each. For each panel, the *x*-axis indicates the percentile of values of the betweenness centrality at time point *t*. The left *y*-axis indicates participants' likelihood of submitting an initial content at time point *t*+1. The right *y*-axis indicates the proportion of initial contents submitted by the participants to all the initial contents in the communities. The red lines corresponding to the right *y*-axis show what percentages of initial contents were submitted by participants with different values of the core-periphery metrics. The blue lines corresponding to the left *y*-axis show what percentages of participants with different values of core-periphery metrics would submit an original content at the next time point. The grey area shows the 95% confidence intervals of the blue lines, generated by two-tailed *t*-tests (note that the confidence intervals for the GitHub data are too narrow to be seen). The insets show the predicted possibilities of initial content submissions by the participants who had different values of betweenness centrality but shared the same number of days in the communities (which equals to the average number days that all participants spent in the communities). The predicted possibilities of initial content submissions were generated by a logistic model with the likelihood of initial content submissions as the dependent variable, the values of the betweenness centrality as the independent variable, and the number of days that a participant spent in the communities as the control variable.

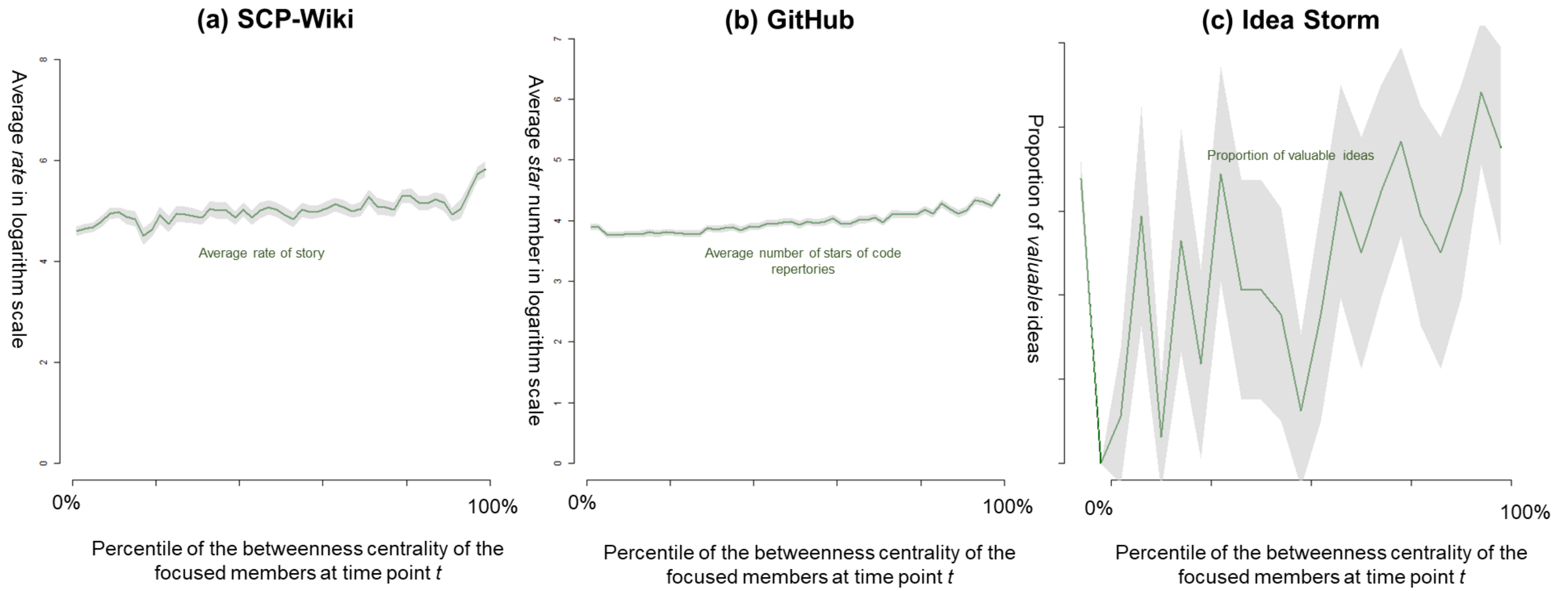

Fig. S7 Illustration of the relationship between the values of the betweenness centrality and the quality of final outcomes; in Fig. S7, the relationships between the different values of the betweenness centrality and the values of the content-quality metrics in SCP-Wiki (a), GitHub (b), and Idea Storm (c) are shown for each. For each panel, the *x*-axis indicates the percentile of values of the betweenness centrality at time point *t*. The *y*-axis indicates the value of each quality metric of the content; in SCP-Wiki data, it represents the average rate on a logarithmic scale; in GitHub data, it represents the average star number on a logarithmic scale; in Idea Storm data, it represents the average proportion of valuable ideas. The green lines show the relationship between originators' values of the betweenness centrality and the average quality of the final outcomes. The grey area shows the 95% confidence intervals of the average value generated by the two-tailed *t*-test (note that the confidential intervals in GitHub data are too narrow to be seen). Note that, in the Idea Storm data, the proportion of valuable ideas fluctuated. This is because most participants only submitted a small number of ideas (e.g., one or two ideas). Specifically, in many cases, even only one idea was evaluated as valuable. Therefore, it inevitably generated a large fluctuation (e.g., from 0 % to 50 %) in the proportion of valuable ideas.

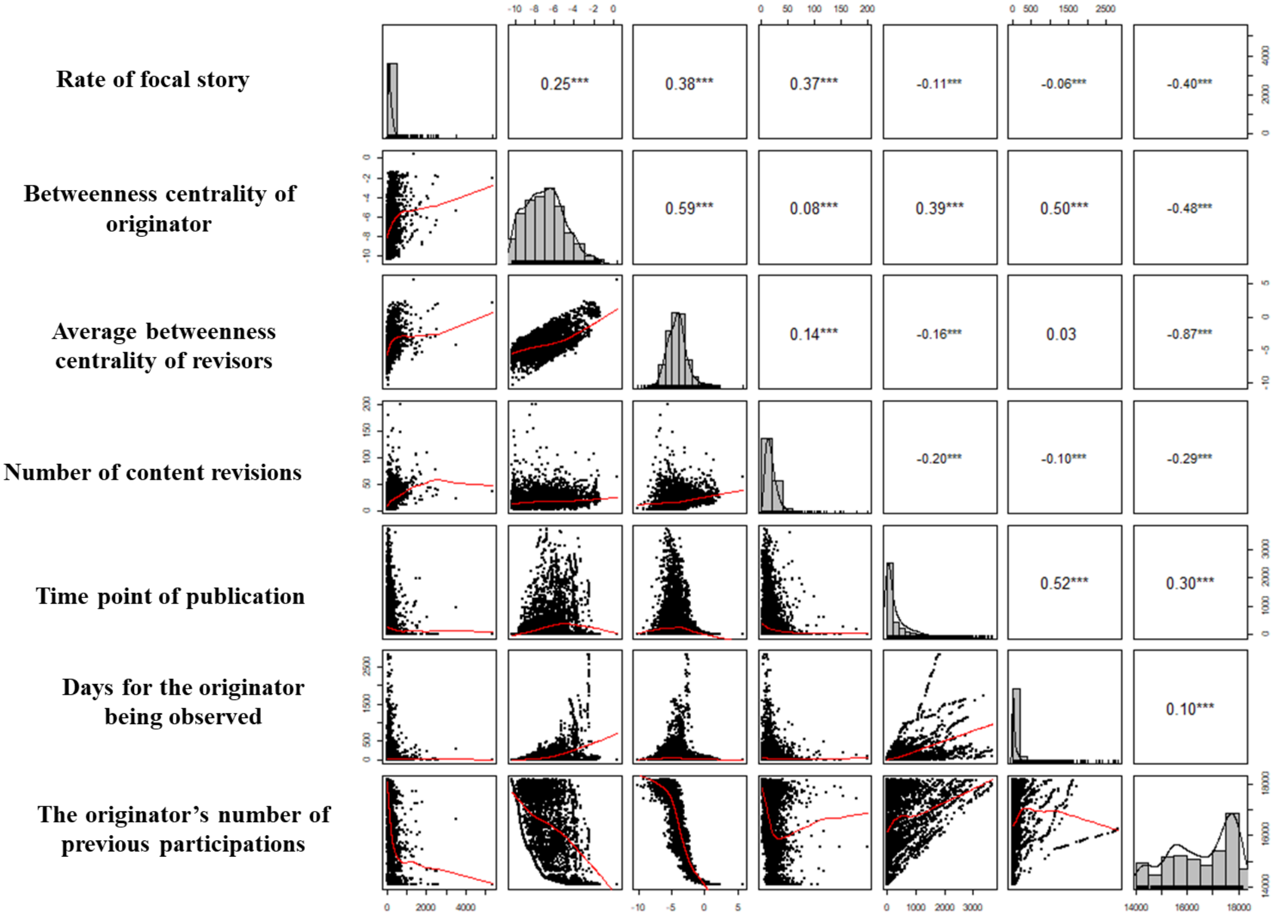

Fig. S8. Distribution, scatter plots, and correlations of all variables in the supplementary regression model of SCP-Wiki content quality; the names of all variables are in the left of the figure. Note that the names of the variables are abridged for display in the figure. In this figure, the diagonal shows histograms with the density curves of each variable in the regression model of content quality in Idea Storm. Graphs in the lower triangle show scatter plots between each pair of variables. The upper triangle presents significant correlations between each pair of variables. One asterisk refers to a *p*-value smaller than 0.1, two asterisks refer to a *p*-value smaller than 0.05, and three asterisks refer to a *p*-value smaller than 0.01.

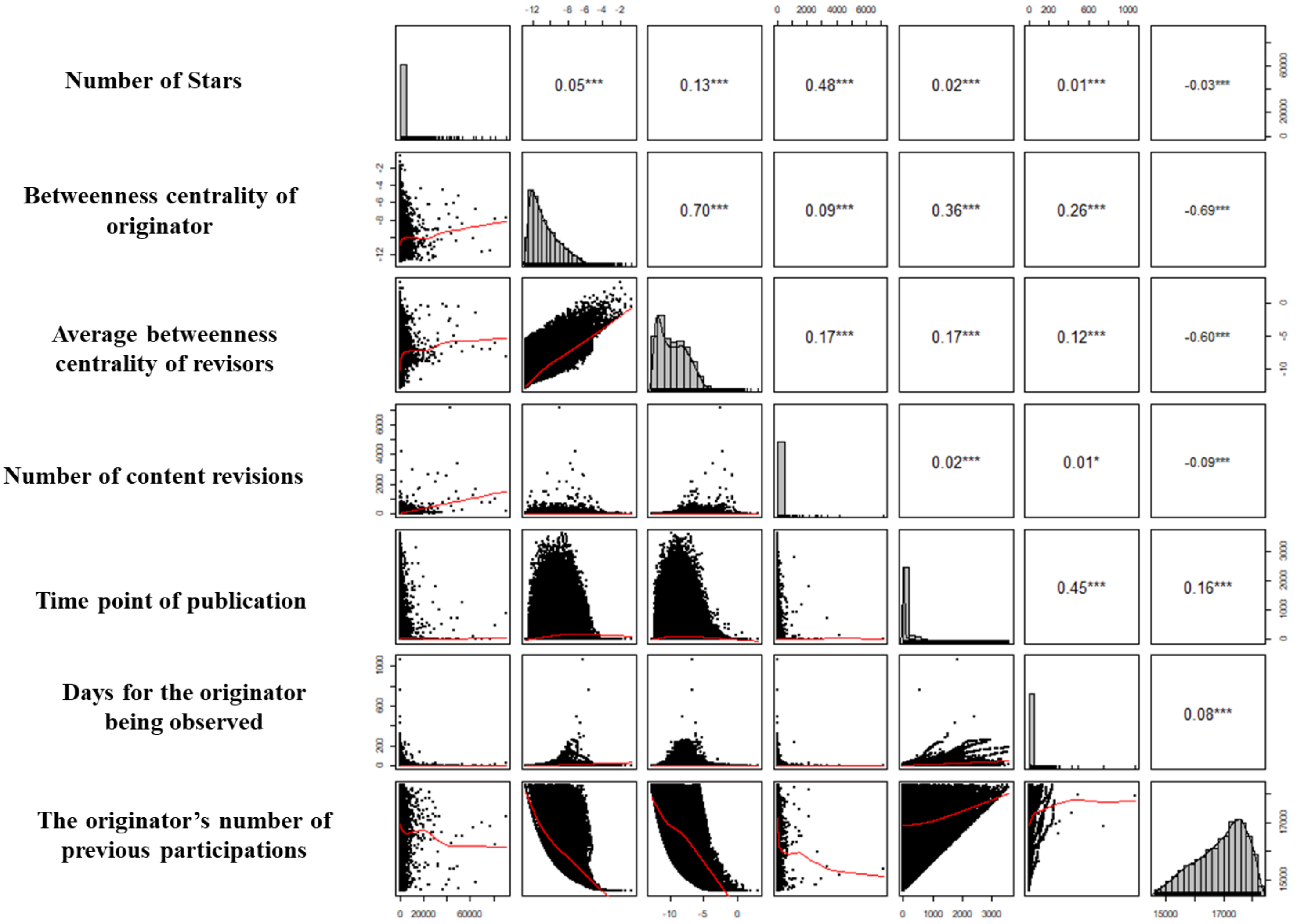

Fig. S9. Distribution, scatter plots, and correlations of all variables in the supplementary regression model of GitHub content quality; the names of all variables are in the left of the figure. Note that the names of the variables are abridged for display in the figure. In this figure, the diagonal shows histograms with the density curves of each variable in the regression model of content quality in Idea Storm. Graphs in the lower triangle show scatter plots between each pair of variables. The upper triangle presents significant correlations between each pair of variables. One asterisk refers to a *p*-value smaller than 0.1, two asterisks refer to a *p*-value smaller than 0.05, and three asterisks refer to a *p*-value smaller than 0.01.

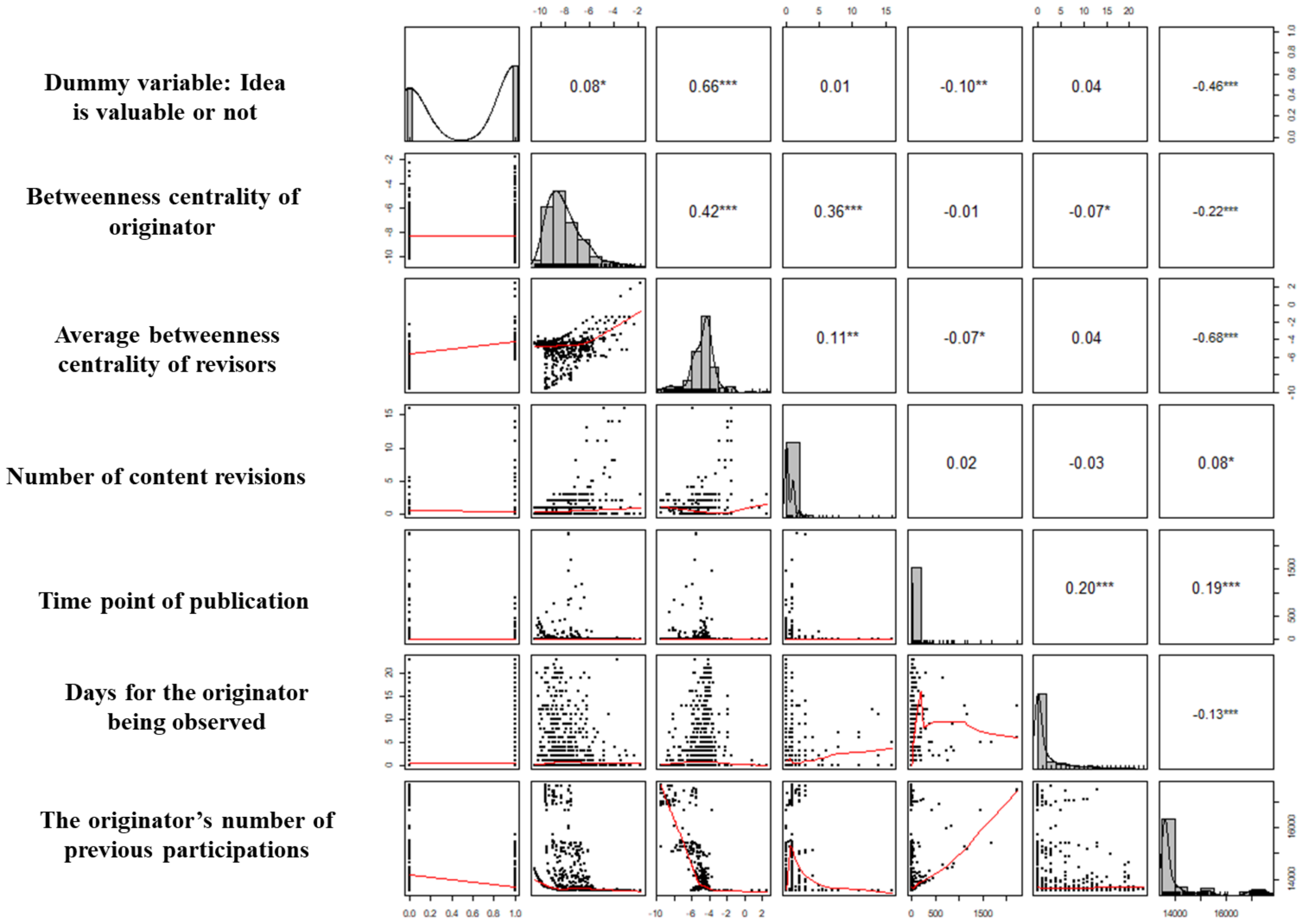

Fig. S10. Distribution, scatter plots, and correlations of all variables in the supplementary regression model of Idea Strom content quality; the names of all variables are in the left of the figure. Note that the names of the variables are abridged for display in the figure. In this figure, the diagonal shows histograms with the density curves of each variable in the regression model of content quality in Idea Storm. Graphs in the lower triangle show scatter plots between each pair of variables. The upper triangle presents significant correlations between each pair of variables. One asterisk refers to a *p*-value smaller than 0.1, two asterisks refer to a *p*-value smaller than 0.05, and three asterisks refer to a *p*-value smaller than 0.01.

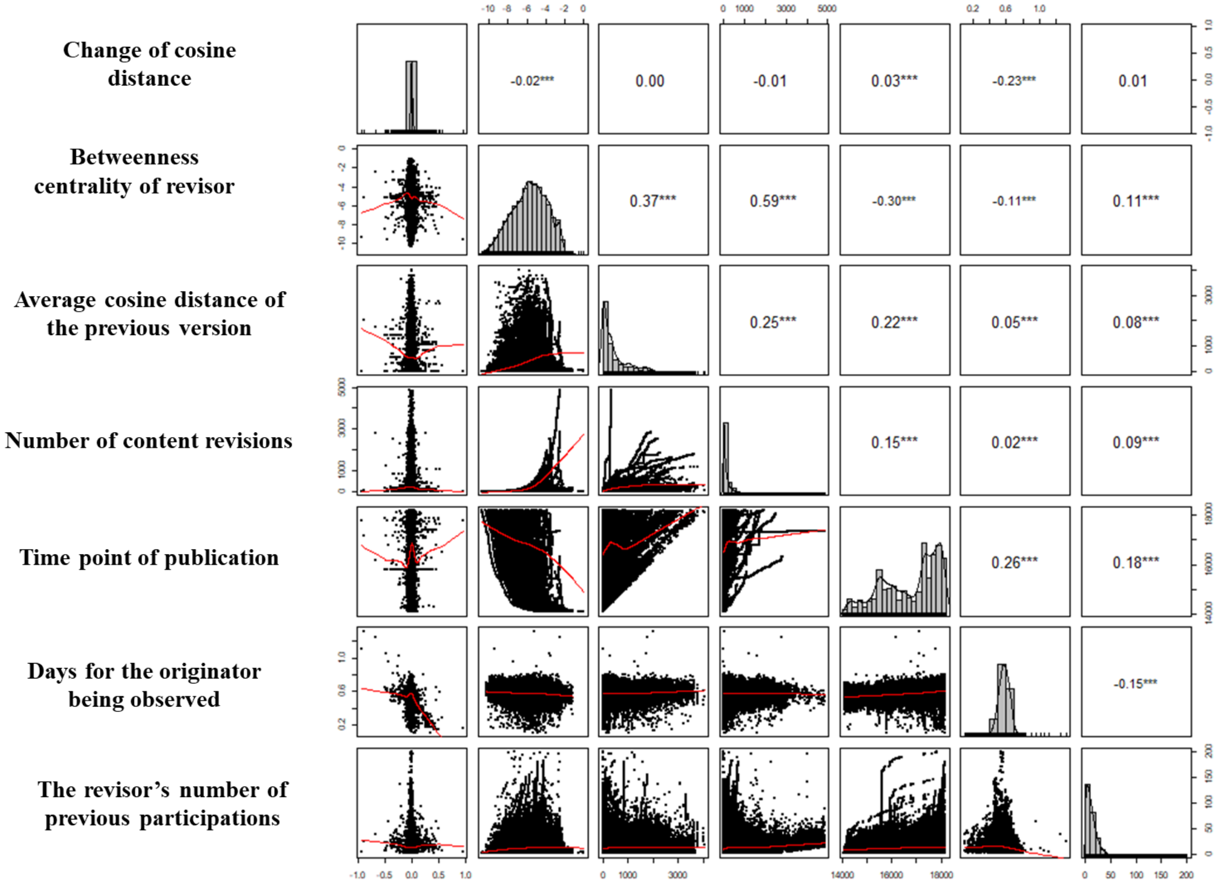

Fig. S11. Distribution, scatter plots, and correlations of variables in the supplementary regression model of originality change; the names of all variables are in the left of the figure. In this figure, the diagonal shows histograms with the density curves of each variable in the regression model of originality change. Graphs in the lower triangle show scatter plots between each pair of variables. The upper triangle presents significant correlations between each pair of variables. One asterisk refers to a *p*-value smaller than 0.1, two asterisks refer to a *p*-value smaller than 0.05, and three asterisks refer to a *p*-value smaller than 0.01.

Table S1. Statistics of variables in regression of the quality of content

**Statistic**	**Observations**	**Mean**	**St. Dev.**	**Min**	**Max**
SCP-Wiki					
Rate of focal story	4,653	180.294	219.974	−20	5,355
Degree of originator	4,653	128.338	275.132	0.000	2,818
K-core of originator	4,653	48.988	89.710	0.000	992
Eigenvector centrality of originator	4,653	0.009	0.057	0.000	0.707
Betweenness centrality of originator	4,653	0.0035	0.036	0.00002	1
Average degree of revisors	4,653	451.296	269.710	2.500	2,566
Average k-core of revisors	4,653	177.704	100.779	1.000	979.700
Average eigenvector centrality of revisors	4,653	0.012	0.034	0.000	0.623
Average betweenness centrality of revisor	4,653	0.090	0.214	0.00001	1
Number of content revisions	4,653	19.629	14.108	2	200
Time point of publication	4,653	16,518.870	1,230.808	14,079	18,174
Days the originator was observed	4,653	467.432	694.090	0.000	3,683.698
The originator’s number of previous participations	4,653	121.719	271.833	0	2,816
GitHub					
Number of Stars	99,235	194.324	1,087.2	5	90,351
Degree of originator	99,235	82.322	435.400	0	35,145
K-core of originator	99,235	9.047	49.415	0	1,728
Eigenvector centrality of originator	99,235	0.0003	0.010	0	0.707
Betweenness centrality of originator	99,235	0.0002	0.0021	0.00001	0.25
Average degree of revisors	99,235	853.071	2,992.168	2	79,727.5
Average k-core of revisors	99,235	21.346	50.298	1	1,368.817
Average eigenvector centrality of revisors	99,235	0.0003	0.004	0.000	0.707

Average betweenness centrality of revisor	99,235	0.0024	0.090	0.00001	0.23
Number of content revisions	99,235	7.962	45.371	1	7,153
Time point of publication	99,235	16,894.690	858.192	14,610	18,300
Days the originator was observed	99,235	276.965	503.548	0.000	3,605
The originator’s number of previous participations	99,235	3.563	15.698	0.000	1,069
Idea Storm					
Dummy variable: Idea is valuable or not	837	0.589	/	0	1
Degree of originator	837	3.907	8.583	0.000	113
K-core of originator	837	2.826	5.713	0.000	37
Eigenvector centrality of originator	837	0.0002	0.002	0.000	0.045
Betweenness centrality of originator	837	0.0009	0.004	0.00001	0.062
Average degree of revisors	837	76.774	73.930	0.000	775.000
Average k-core of revisors	837	36.614	34.130	0.000	358.000
Average eigenvector centrality of revisors	837	0.027	0.024	0.000	0.296
Average betweenness centrality of revisor	837	0.089	0.893	0.00001	0.143
Number of content revisions	837	0.742	1.661	0	16
Time point of publication	837	14,194.400	1,143.889	13,571	17,660
Days the originator was observed	837	33.227	165.736	0	2,240
The originator’s number of previous participations	837	2.804	4.955	0	23

Table S2. Statistic of variables in regression of the change of originality.

Statistic	Observations	Mean	St. Dev.	Min	Max
Dependent Variables					
Change of cosine distance	43,693	0.0002	0.030	−0.935	0.957
Independent Variables					
Degree of revisor	43,693	511.160	676.245	1	4,868
K-core of revisor	43,693	222.524	347.891	0.000	1,896
Eigenvector centrality of revisor	43,693	0.019	0.072	0.000	1
Betweenness centrality of revisor	43,693	0.014	0.02	0.00002	1
Control Variables					
Average cosine distance of the previous version	43,693	0.573	0.072	0.107	1.317
Number of content revisions	43,693	16.278	13.482	2	200
Time point of publication	43,693	16,698.410	1,125.040	14,085	18,175
Days the revisor was observed	43,693	655.539	718.230	0.000	4,025.694
The revisor’s number of previous participations	43,693	464.909	684.150	0	4,866

Table S3. The supplementary results of the regression of content quality for SCP-Wiki data; the coefficients of the core-periphery metrics (i.e., the independent variable) is shown in the fourth row.

**Dependent Variable: Rate in logarithm scale in SCP-Wiki**	
**Core-periphery metric: Betweenness centrality**	
Variable	Coefficient
Betweenness centrality of originator in logarithm scale	0.008 (0.021)
Average betweenness centrality of revisors in logarithm scale	0.245^{***} (0.030)
Number of content revisions	0.304^{***} (0.014)
Time point of publication	-0.05^{*} (0.029)
Days the originator was observed	0.005 (0.018)
The originator's number of previous participations	-0.072^{**} (0.017)
Constant	-0.000 (0.013)
R^2	0.250
Observations: 4,653	

Note: $^{*}p < 0.1$; $^{**}p < 0.05$; $^{***}p < 0.01$; Standard Error shown in ( ); since all coefficients in the table were estimated based on the standardized variables, the sizes of the coefficients are comparable.

Table S4. The supplementary results of the regression of content quality for GitHub data; the coefficients of the core-periphery metrics (i.e., the independent variable) is shown in the fourth row.

**Dependent Variable: Number of stars in logarithm scale in GitHub**	
**Core-periphery metric: Betweenness centrality**	
Variable	Coefficient
Betweenness centrality of originator in logarithm scale	-0.018*** (0.006)
Average betweenness centrality of revisors in logarithm scale	0.102*** (0.004)
Number of content revisions	0.475*** (0.003)
Time point of publication	0.062*** (0.005)
Days the originator was observed	-0.006 (0.004)
The originator's number of previous participations	−0.002 (0.003)
Constant	0.000 (0.003)
R^2	0.241
Observations: 99,232	

Note: $^{*}p < 0.1$; $^{**}p < 0.05$; $^{***}p < 0.01$; Standard Error shown in ( ); since all coefficients in the table were estimated based on the standardized variables, the sizes of the coefficients are comparable.

Table S5. The supplementary results of the regression of content quality for Idea Strom data; the coefficients of the core-periphery metrics (i.e., the independent variable) is shown in the fourth row.

**Dependent Variable: Idea is valuable or not in in Idea Storm**	
**Core-periphery metric: Betweenness centrality**	
Variable	Coefficient
Betweenness centrality of originator in logarithm scale	-0.421*** (0.149)
Average betweenness centrality of revisors in logarithm scale	5.775*** (0.433)
Number of content revisions	0.365* (0.190)
Time point of publication	0.279 (0.187)
Days the originator was observed	-0.343* (0.190)
The originator's number of previous participations	−0.039 (0.123)
Constant	0.505*** (0.129)
AIC	449.211
Observations: 837	

Note: $^{*}p < 0.1$; $^{**}p < 0.05$; $^{***}p < 0.01$; Standard Error shown in ( ); since all coefficients in the table were estimated based on the standardized variables, the sizes of the coefficients are comparable.

Table S6. The supplementary results of the regression on the change of originality for SCP-Wiki data

Dependent Variable: Change of cosine distance among SCP-Wiki drafts	
**Core-periphery metric: Betweenness centrality**	
Variable	Coefficient
Betweenness centrality of originator in logarithm scale	−0.018** (0.007)
Average cosine distance of the previous version	−1.88*** (0.01)
Number of content revisions	0.06*** (0.010)
Time point of publication	−0.02*** (0.008)
Days the revisor was observed	−0.012** (0.004)
Revisor's number of previous participations	0.008 (0.006)
Average constant#	−0.104
R^2	0.440
Observations: 43,693	

Note: *p < 0.1; **p < 0.05; ***p < 0.01; Standard Error shown in ( ); all coefficients in the table were estimated based on the standardized variables. Thus, the sizes of the coefficients are comparable. Since the regression estimated a different constant for each SCP-story, there were 4,653 different constants. For simplification, we report the average constant here.
